# Fermentation Enhances Antioxidant, Antiplatelet, and Anti-Inflammatory Properties of Oat- and Soy-Derived Dairy Alternatives

**DOI:** 10.3390/nu18081260

**Published:** 2026-04-16

**Authors:** Nikolaos Koutis, Georgios Liepouris, Ilianna Moysidou, Lydia Vogiatzaki, Katie Shiels, Sushanta Kumar Saha, Anna Ofrydopoulou, Alexandros Tsoupras

**Affiliations:** 1 Hephaestus Laboratory, School of Chemistry, Faculty of Sciences, Democritus University of Thrace, 65404 Kavala, Greece; nikouti@chem.duth.gr (N.K.); geliepo@chem.duth.gr (G.L.); ilsidou@chem.duth.gr (I.M.); lyvogia@chem.duth.gr (L.V.); anofrid@chem.duth.gr (A.O.); 2 Centre for Applied Bioscience Research, Technological University of the Shannon: Midlands Midwest, Moylish Park, V94 E8YF Limerick, Ireland; katie.shiels@tus.ie (K.S.); sushanta.saha@tus.ie (S.K.S.)

**Keywords:** soy, oat, dairy alternatives, antioxidant, anti-inflammatory, PUFA, phenolics, carotenoids, polar lipids, PAF

## Abstract

Background: The increasing demand for plant-based dairy alternatives has stimulated interest in their potential health-promoting properties, particularly when combined with fermentation processes that may enhance the bio-efficacy and bioavailability of bioactive compounds. Methods: The present study investigated the impact of fermentation on the antioxidant, antiplatelet, and anti-inflammatory activities of oat- and soy-based dairy alternatives. Total lipids were extracted and fractionated into lipophilic and amphiphilic lipid fractions, which were subsequently evaluated for antioxidant capacity using 2,2′-azino-bis(3-ethylbenzothiazoline-6-sulfonic acid) (ABTS), 2,2-diphenyl-1-picrylhydrazyl (DPPH), and ferric reducing antioxidant power (FRAP) assays, as well as for their inhibitory activity against platelet aggregation induced by platelet-activating factor (PAF) or by ADP. Results: Fermentation significantly enhanced the biological activity of the tested products, with fermented samples exhibiting lower IC_50_ values and thus more potent anti-inflammatory and antiplatelet efficacy and improved antioxidant performance compared with the non-fermented plant-based dairy alternative products. The amphiphilic lipid fractions demonstrated the strongest bioactivity, suggesting that fermentation promotes structural modifications in polar lipids that contribute to enhanced functional properties. Overall, fermented soy products exhibited stronger antiplatelet (anti-ADP) and anti-inflammatory (anti-PAF) activities, with lower IC_50_ values (indicating higher inhibitory potency), whereas fermented oat products demonstrated particularly enhanced antioxidant capacity, especially in TAC fractions, as evidenced by higher FRAP values and carotenoid content (e.g., oat yogurt TAC: 19.14 ± 9.97 mg CE/g extract). In DPPH assays, TAC fractions of both soy and oat showed comparable radical scavenging activity (TEAC ≈ 0.019 for soy yogurt TAC), while ABTS and FRAP assays highlighted matrix-dependent differences between lipid fractions. Fatty acid analysis further indicated favorable compositional changes associated with fermentation, including favorable alterations in the *n*-6/*n*-3 fatty acid ratio of the fatty acid content of the bioactive polar lipid species, while OMICs analysis indicated the specific molecular species of phospho-/glyco-based polar lipids present in these products. Conclusions: These findings suggest that fermentation can substantially improve the biofunctional profile of plant-based dairy alternatives and highlight fermented oat- and soy-based products as promising dietary sources of bioactive polar lipids with potential cardioprotective properties.

## 1. Introduction

Diet is a fundamental determinant of human health and a major modifiable factor influencing the development of non-communicable diseases (NCDs) [1,2]. According to the World Health Organization, cardiovascular diseases, diabetes, cancer, and chronic respiratory disorders remain leading causes of mortality worldwide, with their prevalence strongly linked to chronic low-grade inflammation and oxidative stress [3,4]. Dietary patterns characterized by excessive intake of energy-dense, nutrient-poor foods and pro-inflammatory profile contribute substantially to thrombo-inflammatory processes, involving potent mediators of inflammation like platelet activating factor (PAF) and other inflammatory cytokines and chemokines, which are involved in platelet and leukocyte activation, and endothelium dysfunction, thereby accelerating the progression of cardiometabolic disorders and tumor onset and development [3,4,5]. Additional lifestyle-related risk factors—including tobacco exposure, alcohol consumption, sedentary behavior, and psychosocial stress—further exacerbate oxidative imbalance and systemic inflammation through such mediators, their signaling, and the subsequent thrombo-inflammatory complications [3,4,5,6,7,8,9].

In response to growing awareness of diet–disease interactions, consumer interest has shifted toward plant-based dietary patterns rich in bioactive constituents with antioxidant and anti-inflammatory properties [10,11]. Plant-derived foods, including dairy alternative drinks and yogurt-style products, seem to provide a diverse array of bioactive molecules, including phenolic compounds, carotenoids, unsaturated fatty acids, vitamins, and polar lipids, many of which have been associated with cardioprotective and antithrombotic effects [12,13,14]. Among all types of plant sources, soybeans occupy the most established position in consumer preferences and are the plant source with the most research from the scientific community for potential health benefits [15]. Concurrently, the increasing prevalence of lactose intolerance and the adoption of vegetarian or vegan dietary practices have stimulated the expansion of plant-based dairy alternatives in global food markets [16,17].

Among plant-based raw materials, soybean (*Glycine max*) has long represented the most extensively studied and commercially established source for dairy substitutes [12,15]. Soy-based beverages and yogurt-type products are characterized by high-quality protein, polyunsaturated fatty acids, phospholipids, and isoflavones such as genistein and daidzein, compounds implicated in lipid regulation, endothelial protection, and modulation of inflammatory biomarkers [18,19,20]. Moreover, lipid-soluble antioxidants such as tocopherols and isoflavons contribute to oxidative stability and functional value [21,22]. Fermentation of soy matrices by lactic acid bacteria (e.g., *Lactobacillus* spp. and *Streptococcus thermophilus*) has been reported to enhance isoflavone bioavailability through enzymatic hydrolysis of glycosidic forms into biologically active aglycones while simultaneously improving sensory and physicochemical properties [23,24,25].

Oat (*Avena sativa*) has emerged as an increasingly prominent alternative plant matrix due to its favorable nutritional profile and functional components [26]. Oats are particularly rich in β-glucans, soluble fibers associated with hypocholesterolemic and glycemic regulatory effects [27]. Additionally, oats contain unique phenolic compounds such as avenanthramides, alongside carotenoids and lipid fractions that may contribute to antioxidant and anti-inflammatory activity [28,29]. Fermentation of oat-based beverages can further modify the bioactive profile by promoting enzymatic release of bound phenolics and enhancing carotenoid bioaccessibility, potentially amplifying biological functionality [30].

Despite the rapid market growth of plant-based dairy alternatives [11,18,19,20], systematic investigations of the lipid-associated bioactive constituents of these dairy alternatives and of several products based on them [31]—particularly amphiphilic and polar lipid fractions—and their functional implications in thromboinflammatory pathways remain limited. While phenolic and carotenoid contents in fruits, vegetables, brands, and their products have been extensively characterized, along with their antioxidant capacities, comparatively fewer studies have examined these compounds within lipid extracts of soy- and oat-derived dairy substitutes, especially in relation to potential antioxidant, anti-inflammatory, and antiplatelet properties against the platelet-activating factor (PAF)-mediated inflammatory signaling. The polar lipid contents of some unfermented and fermented products from almond, rice, and coconut have previously been assessed against PAF and ADP in human platelets with promising results for their fermented products as functional anti-inflammatory foods [14], but not for oat and soy unfermented and fermented dairy alternatives.

The objective of the present study was to investigate the impact of fermentation on the biofunctional properties of the amphiphilic compounds present in plant-based dairy alternatives derived from *A. sativa* (oat) and *G. max* (soy). Specifically, the study aimed to evaluate how fermentation influences the antioxidant, antiplatelet, and anti-inflammatory activities of amphiphilic fractions isolated from commercial oat- and soy-based beverages and their fermented counterparts. To achieve this, total lipids were extracted and separated into lipophilic and amphiphilic fractions, which were subsequently assessed for their capacity to inhibit platelet aggregation induced by either an inflammatory mediator, platelet-activating factor (PAF), or by a classic platelet agonist, adenosine-5′-diphosphate (ADP), as well as for their antioxidant potential. In addition, the fatty acid composition of the polar lipid fractions and LC-MS-based OMICs structural characterization analysis were also carried out in order to explore potential structure–activity relationships of the present polar lipids in these fermented products versus the unfermented ones.

## 2. Materials and Methods

### 2.1. Materials, Reagents, and Instrumentation

Commercial plant-based dairy alternatives derived from *G. max* (soy) and *A. sativa* (oat) were purchased from retail supermarkets in the Kavala region (Greece). The study included four product categories: non-fermented soy beverages, non-fermented oat beverages, fermented soy yogurt-type products, and fermented oat yogurt-type products. For each category, three independent commercial samples (*n* = 3) were analyzed. All products were stored under refrigeration according to the manufacturer’s instructions and analyzed within their declared shelf-life period. All products contained typical formulation components, such as 80–90% water, 8–15% plant extract, stabilizers (e.g., gellan gum), emulsifiers (i.e., plant-derived lecithin), added sugars, and fortification with vitamins (e.g., B12, D) and minerals (e.g., calcium carbonate), which are commonly used in commercial plant-based dairy alternatives. Regarding fermentation cultures, the products analyzed were commercial fermented yogurt-type products; therefore, the exact microbial strains were not explicitly disclosed by the manufacturers. However, according to the product labels and typical industrial practice, the fermentation process was conducted using lactic acid bacteria cultures commonly employed in plant-based yogurt production, primarily *Lactobacillus* spp. and *S. thermophilus.*

Analytical-grade solvents used for extraction and analysis, including methanol (99.8%), ethanol (96.6%), hexane, petroleum ether, n-octane, and chloroform (99.8%), were obtained from Sigma-Aldrich (St. Louis, MO, USA). Reagents used for antioxidant assays included 2,2-diphenyl-1-picrylhydrazyl (DPPH), 2,2′-azino-bis(3-ethylbenzothiazoline-6-sulfonic acid) (ABTS), sodium persulfate, sodium carbonate (Na_2_CO_3_), acetic acid, sodium acetate, Tris(hydroxymethyl)aminomethane (Tris), and hydrochloric acid (HCl). Reference standards such as gallic acid, catechin, quercetin, β-carotene, soybean phospholipids, and Trolox were also supplied by Sigma-Aldrich.

Solvent removal was performed using a Büchi rotary evaporator (Büchi Labortechnik AG, Flawil, Switzerland). Spectrophotometric analyses were conducted with a uniSPEC 2 UV–Vis spectrophotometer (LLG Labware, Meckenheim, Germany). Structural characterization of selected extracts was performed using attenuated total reflectance Fourier-transform infrared spectroscopy (ATR–FTIR) with a Frontier FT-IR spectrometer (PerkinElmer, Waltham, MA, USA).

Platelet aggregation experiments were conducted using human platelet-rich plasma (hPRP) obtained from healthy adult volunteers. Blood samples were collected by venipuncture into sodium citrate vacuum tubes (Sarstedt S-Monovette^®^, Wexford, Ireland) using sterile safety needles. Participants were healthy adults with no known cardiovascular disease and were not receiving medication known to affect platelet function. Donors had not consumed oat- or soy-based products prior to blood collection, minimizing potential dietary interference with platelet responsiveness.

The study protocol was approved by the Ethics Committee of Democritus University of Thrace (protocol code ΔΠΘ/ΕHΔΕ/7690/70; 27 September 2024). Written informed consent was obtained from all volunteers prior to blood collection.

Platelet-rich plasma was prepared by centrifugation of whole blood at low speed to separate platelets from erythrocytes and leukocytes. Platelet aggregation assays were performed using a Chrono-Log Model 490 optical platelet aggregometer (Chrono-Log Corp., Havertown, PA, USA) equipped with AGGRO/LINK^®^ 8 software (Version 8, Chrono-log Corp., Havertown, PA, USA). All measurements were performed under standardized experimental conditions.

### 2.2. Lipid Extraction and Fractionation

Total lipids were extracted from the plant-based samples using a modified chloroform–methanol extraction protocol widely applied for lipid isolation from food matrices, according to Tsoupras et al. 2024 [32]. For each product type, three independent samples were analyzed in triplicate.

Approximately 30 mL of beverage samples or 30 g of yogurt-type products were homogenized prior to extraction. Lipids were initially extracted using a monophasic chloroform/methanol/water mixture (1:2:0.8, *v*/*v*/*v*), which facilitates the disruption of the food matrix and the solubilization of both polar and non-polar lipid components. For semi-solid fermented products, homogenized samples were filtered under vacuum to remove residual solid particles.

Phase separation was subsequently induced by adjusting the solvent ratio to chloroform/methanol/water (1:1:0.9, *v*/*v*/*v*), resulting in the formation of a biphasic system. The lower organic phase containing the lipid fraction was collected and evaporated under reduced pressure at 37 °C using a rotary evaporator.

The resulting total lipid extracts were redissolved in chloroform/methanol (1:1, *v*/*v*) and stored refrigerated under light-protected conditions until further analysis. The extraction yield was calculated as the percentage of lipid mass recovered relative to the initial sample mass.

Extraction Yield (%) = (mass of dry extract (g)/mass of initial sample (g)) × 100(1)

where mass of dry extract = the weight of the lipid residue after solvent evaporation, mass of initial sample = the weight of the oat or soy beverage/yogurt before extraction.

Total lipid extracts were further separated into lipophilic and amphiphilic fractions using a modified countercurrent distribution procedure previously applied to complex food lipid matrices [14,32]. In this approach, hexane was used as the non-polar solvent instead of petroleum ether in order to comply with current European recommendations regarding solvent selection for food-related applications.

This procedure allowed the separation of Total lipophilic lipid fractions, mainly containing neutral lipids and highly hydrophobic compounds, and total amphiphilic lipid fractions, rich in polar lipids such as phospholipids and glycolipids, and other amphiphilic compounds like carotenoids and phenolics.

Following separation, lipophilic fractions were dissolved in hexane, whereas amphiphilic fractions were dissolved in ethanol for further biochemical and biological analyses.

### 2.3. Total Phenolic Content (TPC)

Total phenolic content was determined using the Folin–Ciocalteu assay with minor modifications [32]. Briefly, 1 mL of distilled water and 1 mL of Folin–Ciocalteu reagent were added to each sample. After 7 min, 3 mL Na_2_CO_3_ solution was added, followed by vortex mixing. Samples were incubated in the dark for 2 h, and absorbance was measured at 765 nm. Results were calculated from a gallic acid calibration curve and expressed as mg gallic acid equivalents (GAE)/g extract.

### 2.4. Total Carotenoid Content (TCC)

Total carotenoids were quantified spectrophotometrically as previously described [32]. Samples were dissolved in 2 mL of hexane, and absorbance was measured at 450 nm. Concentrations were calculated using a β-carotene standard curve and expressed as mg β-carotene equivalents (CE)/g extract.

### 2.5. Total Antioxidant Activity (TAA)

The total antioxidant activity (TAA) of TAC and TLC fractions was evaluated using three complementary spectrophotometric assays: the ABTS radical cation decolorization assay, the DPPH radical scavenging assay, and the ferric reducing antioxidant power (FRAP) assay, as described by Tsoupras et al. [32].

For the ABTS assay, 2 mL of pre-formed ABTS•^+^ solution was added to each sample and mixed thoroughly using a vortex. After incubation in the dark for 7 min, absorbance was measured at 734 nm. Trolox was used as the reference antioxidant to generate a calibration curve within an absorbance range of 0.2–0.8. Results were expressed as μmol Trolox equivalents per gram of dry weight (μmol TE/g DW) according to the equation:

ABTS (μmol TE/g DW) = c × V × t/m,(2)

where c represents the concentration determined from the Trolox calibration curve (μmol/mL), V is the sample volume (mL), t is the dilution factor, and m is the mass of the dry extract (g).

For the DPPH assay, samples were mixed with ethanol, distilled water, and DPPH solution, vortexed after each addition, and incubated at room temperature for 30 min. Absorbance was recorded at 517 nm against an appropriate blank. Radical scavenging activity (%) was calculated as follows:

% inhibition = [(A_1_ − A_2_)/A_1_] × 100, (3)

where A_1_ is the absorbance of the control, and A_2_ is the absorbance in the presence of the extract. The IC_50_ value (concentration required to inhibit 50% of DPPH radicals) was calculated, and antioxidant capacity was expressed as Trolox equivalent antioxidant capacity (TEAC):

TEAC = IC_50_(Trolox)/IC_50_(sample). (4)

For the FRAP assay, 3 mL of FRAP reagent was added to each sample, vortexed, and incubated in the dark for 15 min. Absorbance was measured at 593 nm. Results were expressed as μmol TE/g DW using the equation:

FRAP (μmol TE/g DW) = c × V × t/m,(5)

where variables are defined as above.

Trolox was used as a positive control and reference antioxidant standard in all antioxidant assays (ABTS, DPPH, and FRAP), allowing results to be expressed as Trolox Equivalent Antioxidant Capacity (TEAC) or μmol TE/g DW. Appropriate blanks (solvent without extract) were used as negative controls to account for background absorbance. All measurements were performed in triplicate to ensure reproducibility.

### 2.6. Evaluation of Fatty Acid Composition by LC–MS

Liquid chromatography–mass spectrometry (LC–MS) was employed to determine the fatty acid composition of the bioactive polar lipids (PLs) of the total amphiphilic compound (TAC) fractions and to characterize the structural profile of these PLs, as previously described by Tsoupras et al. [32].

Dried extracts were reconstituted in 500 μL dichloromethane/methanol (1:2, *v*/*v*), centrifuged at 13,000 rpm for 6 min (Heraeus Biofuge Stratos, Fisher Scientific, Dublin, Ireland), and the supernatants were filtered through 0.2 μm PTFE syringe filters (Sartorius, Göttingen, Germany). An aliquot (10 μL) of each filtrate was injected into an Agilent 1260 HPLC system coupled to an Agilent 6520 Q-TOF mass spectrometer (Agilent Technologies, Santa Clara, CA, USA) equipped with an electrospray ionization (ESI) source.

Chromatographic separation was achieved on a Poroshell 120 EC-C18 column (Agilent Technologies, Santa Clara, CA, USA) (2.7 μm, 3.0 × 150 mm) using gradient elution with mobile phase A (2 mM ammonium acetate in water) and mobile phase B (2 mM ammonium acetate in 95% acetonitrile). The flow rate was set at 0.3 mL/min (0–5 min), increased to 0.6 mL/min at 10 min, and maintained until completion of the run. Mass spectra were acquired in negative ion mode over an *m*/*z* range of 50–1100, with reference masses (*m*/*z* 1033.988 and 112.9855) for internal calibration. Instrument parameters included a capillary voltage of 3500 V, skimmer voltage of 65 V, fragmentor voltage of 175 V, drying gas flow of 5 L/min, nebulizer pressure of 30 psi, and drying gas temperature of 325 °C.

Method validation was performed by comparing exact masses and retention times with authenticated standards of saturated and unsaturated fatty acids (C12:0–C22:6). Relative fatty acid abundance was calculated from the mean peak areas of triplicate injections. Structural assignment of free fatty acids and polar lipid species (phospholipids and glycolipids) was based on MS^1^ scans, fragmentation patterns, precursor and product ion analysis, and neutral loss profiling. Identifications were further confirmed using the LIPID MAPS database in conjunction with fatty acid profiles of saponified PLs, as previously reported [30].

### 2.7. Evaluation of the Antiplatelet and Anti-Inflammatory Properties of Extracts by Cumulative Light Transmission Measurements

The antiplatelet and anti-inflammatory properties of TAC and TLC fractions derived from soy and oat beverages and their corresponding yogurt-type products were assessed using light transmission aggregometry (LTA) in human platelet-rich plasma (hPRP) obtained from healthy donors, as previously described [33].

The inhibitory capacity of each extract was evaluated against platelet aggregation induced by platelet-activating factor (PAF), a key inflammatory and thrombotic mediator, as well as by adenosine diphosphate (ADP), a classical platelet agonist. In selected experiments, thrombin receptor-activating peptide (TRAP) was also used to assess antithrombotic activity. Platelet aggregation was monitored as changes in light transmittance: resting hPRP exhibits low light transmission, whereas agonist-induced platelet aggregation increases light permeability proportionally to the extent of aggregation.

Extracts were tested at multiple concentrations to generate concentration–response curves within the linear range of 20–80% of maximum reversible aggregation. The half-maximal inhibitory concentration (IC_50_) was calculated from the linear regression of percent inhibition versus extract concentration. IC_50_ represents the mass (μg) of dry extract required to inhibit 50% of agonist-induced platelet aggregation. Lower IC_50_ values indicate greater inhibitory potency against the specific aggregation pathway.

Results were expressed as IC_50_ values for each standard platelet agonist (PAF and ADP), which served as reference stimuli in the platelet aggregation experiments. The inhibitory capacity of each extract was evaluated against platelet aggregation induced by PAF and ADP, which served as positive controls for platelet activation. Baseline platelet-rich plasma (hPRP) without agonist stimulation was used as the negative control, representing 0% aggregation.

Extracts were tested at multiple concentrations to generate concentration–response curves within the linear range of 20–80% of maximum reversible aggregation. The half-maximal inhibitory concentration (IC_50_) was calculated from the linear regression of percent inhibition versus extract concentration. Lower IC_50_ values indicate greater inhibitory potency.

All experiments were performed in independent replicates using platelets from different donors (*n* = 6 different blood donors’ samples for each TAC fraction, thus 3 × 6 = 18 different IC_50_ values for the triplicate TAC fractions from a plant-based sample) to ensure reproducibility. Inclusion criteria, including healthy adult donors with no history of cardiovascular disease or medication affecting platelet function, along with clarifications that blood donors served as biological sources for platelet-rich plasma, while the experimental comparisons were performed between the tested lipid extracts and the platelet agonists used as controls, are embedded in the Informed Consent Form, which was provided to all healthy blood donors involved in the study prior to their participation; the signed form was obtained from all these participants for being allowed to participate, according to the Ethics Statement from the Ethics Committee of Democritus University of Thrace (protocol code: ΔΠΘ/ΕHΔΕ/7690/70, approval date: 27 September 2024).

### 2.8. ATR–FTIR Structural Analysis

Structural characterization of the extracts was performed using attenuated total reflection Fourier-transform infrared spectroscopy (ATR–FTIR), as previously described [32]. In this technique, infrared radiation is directed through an internal reflection crystal of high refractive index. When the sample is placed in direct contact with the crystal surface, an evanescent wave penetrates a few micrometers into the material. Absorption of specific wavelengths induces vibrational transitions in molecular bonds, generating a characteristic infrared spectrum that reflects functional groups and overall molecular structure.

ATR–FTIR analysis was conducted using a PerkinElmer Frontier ATR/FT-NIR/MIR spectrophotometer (PerkinElmer, Shelton, CT, USA) within the spectral range of 600–4000 cm^−1^. A small quantity of each extract was applied directly onto the crystal surface to ensure full coverage and optimal contact. Spectra were recorded once stable signal acquisition was achieved.

All TAC and TLC extracts were analyzed under identical conditions. Reference spectra were also obtained for analytical standards (quercetin, catechin, gallic acid, β-carotene, and polar soybean lipids) to facilitate peak assignment and comparative interpretation. Isopropanol, used for crystal cleaning between measurements, was additionally analyzed to identify and exclude potential solvent-related spectral interference. The ATR–FTIR technique was selected due to its minimal sample preparation requirements, low sample volume demand, and non-destructive nature.

### 2.9. Statistical Analysis

Statistical analysis was performed using IBM SPSS Statistics for Windows, Version 29.0 (IBM Corp., Armonk, NY, USA). Data normality was assessed using the Shapiro–Wilk test. For normally distributed variables, differences among groups were evaluated using one-way analysis of variance (ANOVA). When the assumption of normality was not met, the non-parametric Kruskal–Wallis test was applied. Statistical significance was defined as *p* < 0.05.

## 3. Results

### 3.1. Yield of Extraction

The extraction yield of all samples, including the total lipid (TL) extracts and their respective subfractions—triacylglycerols (TAC) and total lipid content (TLC)—was quantified and expressed as grams of extract per 100 g of sample (g extract/100 g sample). The analyzed matrices comprised three soy-derived non-fermented dairy alternative drinks (1SG, 2SG, and 3SG), three oat-derived non-fermented dairy alternative drinks (1ΒG, 2ΒG, and 3ΒG), three soy-derived fermented dairy alternative yogurt-type products (1Sg, 2Sg, and 3Sg), and three oat-derived fermented dairy alternative yogurt-type products (1Βg, 2Βg, and 3Βg). Given that a fixed volume of 30 mL of each non-fermented dairy alternative drink was subjected to extraction, the natural density of each sample was employed to convert volume to mass, thereby facilitating standardized yield calculations. Based on the findings of T. Daszkiewicz et al. [34], the densities of soy and oat beverages were determined to be 1.0133 g/mL and 1.0254 g/mL, respectively.

Analysis of the extraction data allows several conclusions to be drawn regarding the quantity and distribution of lipid fractions in soy- and oat-based dairy alternatives. Across all product categories, the yield of total amphiphilic compounds (TAC) consistently exceeded that of total lipophilic compounds (TLC), indicating a predominance of amphiphilic lipid species in these matrices.

Moreover, fermented products exhibited higher total lipid (TL) contents compared with their corresponding non-fermented dairy alternative drinks. Specifically, soy-derived yogurt-type fermented dairy alternative yielded an average of 4.4603 g TL/100 g sample, whereas the soy-derived non-fermented dairy alternative drink (beverage) yielded 3.8410 g TL/100 g sample as depicted in Table 1. A similar trend was observed in oat-based products, where oat yogurt contained substantially higher TL levels (6.3064 g TL/100 g sample) than the oat beverage (3.6430 g TL/100 g sample). These findings suggest that fermentation may enhance lipid extractability, potentially through structural modifications of the matrix or the release of lipid components previously bound to macromolecules.

**Figure 1:**
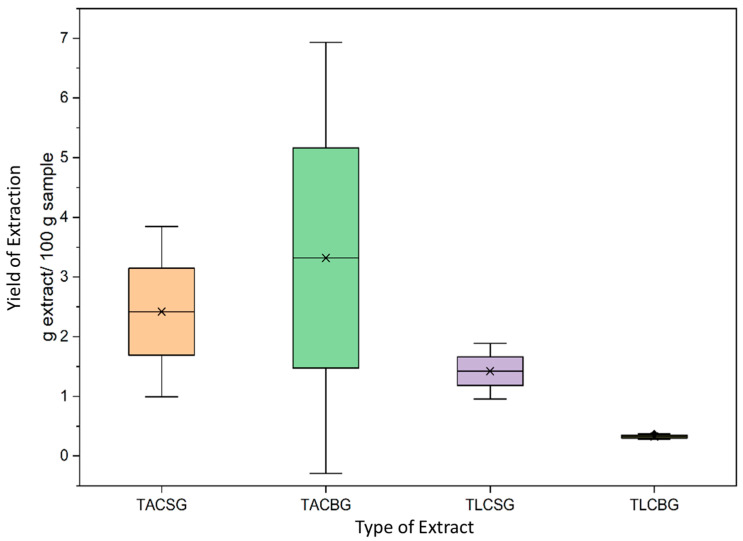
Extraction yields in soybean and oat beverages (*n* = 3). Results are expressed as means with standard deviation. Abbreviations: TACSG: amphiphilic lipids in soybean beverage; TACBG: amphiphilic lipids in oat beverage; TLCSG: lipophilic lipids of soy beverage; TLCBG: lipophilic lipids of oat beverage.

**Figure 2:**
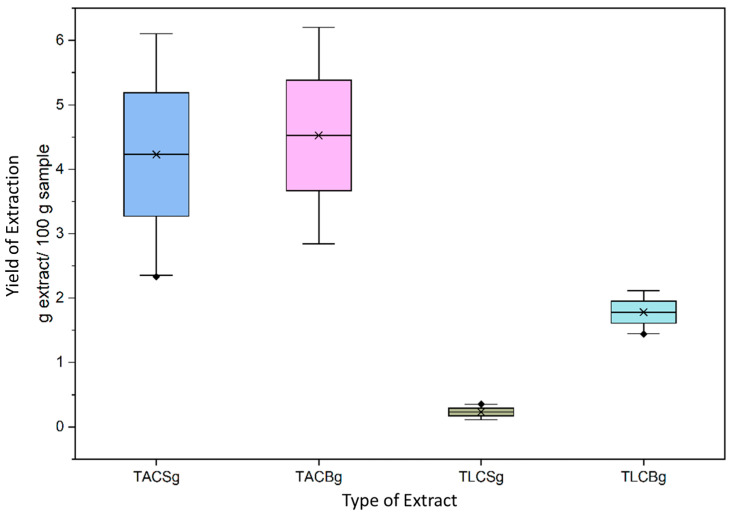
Extraction yield in soybean and oat plant yogurts (*n* = 3). Results are expressed as means with standard deviations. Abbreviations: TACSg: amphiphilic lipids in soybean drink; TACBg: amphiphilic lipids in oat drink; TLCSg: lipophilic lipids of soy beverage; TLCBg: lipophilic lipids of oat beverage.

Although the overall TL content was comparable between soy- and oat-based beverages, marked differences were observed in the distribution of lipid fractions in Figure 1 and Figure 2. In the oat beverage, TAC levels (3.3183 g/100 g sample) were approximately one order of magnitude higher than TLC levels (0.3247 g/100 g sample), demonstrating a pronounced predominance of amphiphilic lipids. In contrast, the soy beverage also showed higher TAC (2.4187 g/100 g sample) than TLC (1.4223 g/100 g sample), but with a less pronounced disparity between fractions.

Given the relatively recent and rapid expansion of plant-based dairy alternatives in the consumer market, the literature addressing standardized methodologies for total lipid extraction in these products remains limited. Notably, Morita et al. [35] reported a TL yield of 3.22 g/100 g sample for soy beverages using the Bligh and Dyer chloroform–methanol extraction method, which is in close agreement with the present findings (3.8410 g/100 g sample), thereby supporting the methodological reliability of the current approach.

Finally, examination of the standard deviation values indicates greater reproducibility and stability in TLC fractions compared with TAC fractions. The consistently higher proportion of TAC lipids across all plant-based products is of particular biochemical interest, as amphiphilic lipid species—such as phospholipids and glycolipids based on lipidomic analysis of such plant-based products [36]—are known to possess significant biological activity, including antioxidant, anti-inflammatory, and antithrombotic properties.

At this point, it is also important to state the importance of the solvents used for the counter-current distribution analyses that facilitated the separation of the TAC from the TLC of all TL extracts. The commonly accepted green solvent ethanol and hexane combination represents an alternative in food lipid extraction protocols; it cannot be considered a fully green solvent, even though it is characterized as such by EU regulations. The requirement that EU states and emphasizes is that even when using such simple solvents, no residue should remain in extracts; thus, a very efficient evaporation is needed to remove them and their extracts from the food stuff, as we indeed performed in this study by elaborating and utilizing a very efficient BUCHI flash rotary evaporator vacuum system (BUCHI 300). Thus, the replacement of petroleum ether with hexane was primarily intended to comply with EU-approved solvent systems commonly used for edible lipid extraction of foodstuffs, rather than to claim complete environmental sustainability. This was also performed as a cost-effective alternative solid-to-solid fast extraction process for such a screening compared to much more expensive methodologies like the supercritical fluid extractions and HPLC analyses [3], which might be more efficient and more appropriate green methodologies for industrial-level separation of lipids. Thus, we used simple, less costly, and still efficient methodologies in such separations, like the current counter distribution based on EU-licensed green solvents, when screening for the first time the bio-efficacy of amphiphilic and lipophilic lipid fractions, prior to more sophisticated analysis, like the LC-MS-based OMICs analysis of the polar lipids we performed as a second stage of analysis in this study.

### 3.2. ATR–FTIR Spectral Characterization of Extracts

Attenuated total reflection–Fourier-transform infrared (ATR–FTIR) spectroscopy was employed as a complementary structural characterization technique to qualitatively assess the major bioactive constituents present in soy- and oat-based products. This approach enabled the identification of functional groups associated with phenolic compounds, polar lipids, and carotenoids, thereby supporting the biochemical findings related to antioxidant and anti-inflammatory activity. Comparative analysis with spectra obtained from reference standards demonstrated substantial spectral similarities between plant extracts and authentic compounds.

**Figure 3:**
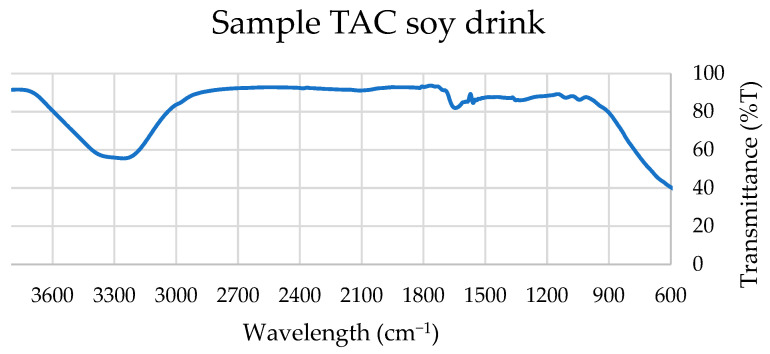
ATR–FTIR spectrum of the most bioactive and richest in amphiphilic bioactive components soybean extract (the *Y*-axis is %Transmittance and the *X*-axis is cm^−1^).

**Figure 4:**
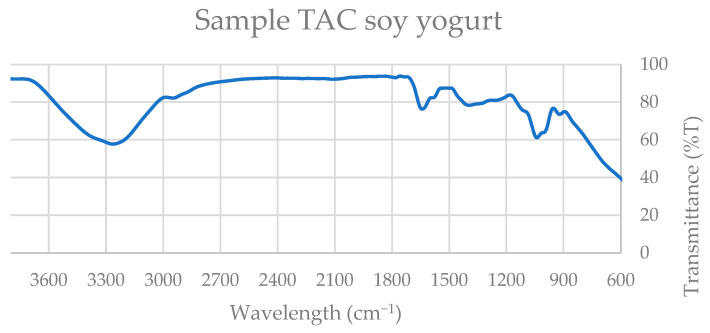
ATR–FTIR spectrum of the most bioactive and richest in amphiphilic bioactive components soy yogurt extract (the *Y*-axis is %Transmittance and the *X*-axis is cm^−1^).

**Figure 5:**
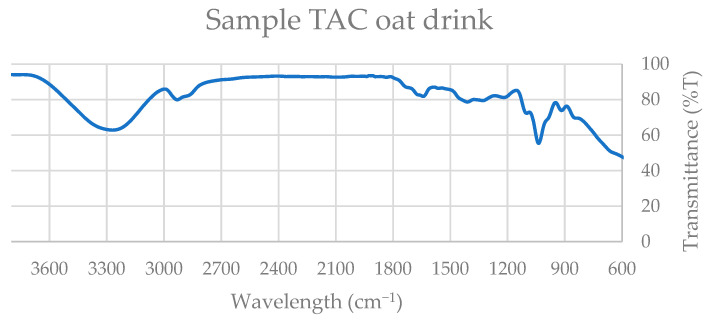
ATR–FTIR spectrum of the most bioactive and richest in amphiphilic bioactive components oat beverage extract (the *Y*-axis is %Transmittance and the *X*-axis is cm^−1^).

**Figure 6:**
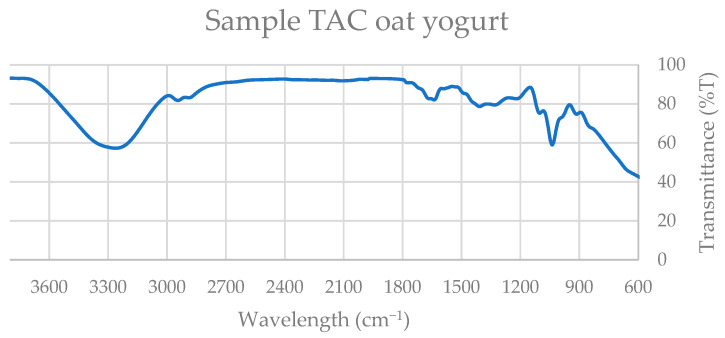
ATR–FTIR spectrum of the most bioactive and richest in amphiphilic bioactive components oat yogurt extract (the *Y*-axis is %Transmittance and the *X*-axis is cm^−1^).

Four representative spectra were selected for detailed evaluation, corresponding to the beverage and yogurt-type formulations of both soy and oat products. The ATR–FTIR spectra (Figure 3, Figure 4, Figure 5 and Figure 6) revealed characteristic absorption bands common across samples and consistent with those observed in the standard compounds (gallic acid, catechin, quercetin, β-carotene, and polar lipid references), as shown in Supplementary Materials (Supplementary Figure S1).

All extracts exhibited a broad absorption band in the 3300–3500 cm^−1^ region, attributable to O–H stretch, a single kinetic step, often present in alcohols, phenols, and carboxylic acids, and is consistent with the presence of phenolic constituents such as gallic acid, catechin, and quercetin.

A distinct absorption band near 2900 cm^−1^, corresponding to C–H stretching vibrations of methyl and methylene groups, was observed in oat products and in soy yogurt, but was absent in the soy beverage. This band is indicative of aliphatic chains found in fatty acids, triglycerides, and carotenoid structures, including β-carotene.

All spectra displayed a narrow band within the 1700–1750 cm^−1^ range, assigned to C=O stretching vibrations of carbonyl groups. This functional group is characteristic of esters, ketones, aldehydes, and carboxylic acids, and is particularly associated with triglycerides and esterified fatty acids in polar lipids.

Additionally, a strong absorption band between 1100 and 1200 cm^−1^ was consistently detected, corresponding to C–O and C–C stretching vibrations. These bands are typical of alcohols, esters, ethers, and phospholipid headgroups, further supporting the presence of amphiphilic lipid species.

Collectively, the spectral features observed at approximately 3400 cm^−1^ (hydroxyl groups), 2900 cm^−1^ (aliphatic C–H stretching), 1700–1750 cm^−1^ (carbonyl groups), and 1100–1200 cm^−1^ (C–O and C–C bonds) confirm the coexistence of phenolic compounds, carotenoids, fatty acids, triglycerides, and polar lipids in the analyzed extracts.

Thus, ATR–FTIR analysis provides structural evidence supporting the biochemical functionality of the samples. The presence of phenolic compounds and carotenoids contributes to the antioxidant potential of the extracts, while the detection of polar lipids and specific flavonoids is consistent with their reported anti-inflammatory and antithrombotic properties.

### 3.3. Evaluation of Phenolic and Carotenoid Content

**Figure 7:**
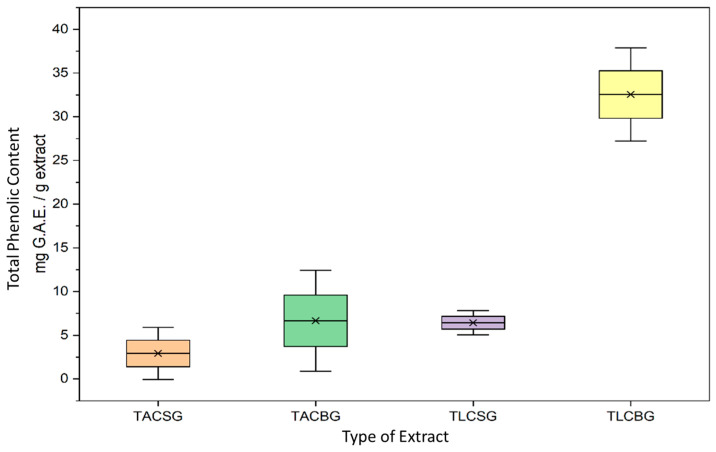
Comparison of total phenolic content in soy and oat beverages, expressed as mg gallic acid equivalent/g extract for total amphiphilic (TAC) and lipophilic compounds (TLC).

**Figure 8:**
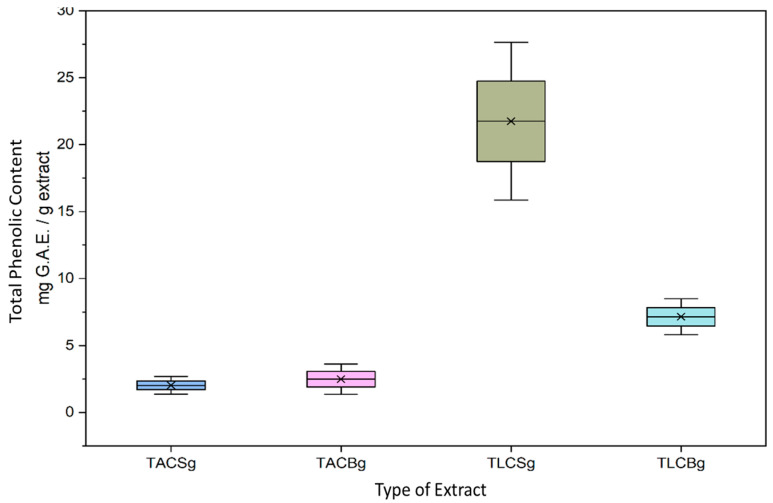
Comparison of total phenolic content in soy and oat yogurts, expressed as mg gallic acid equivalent/g extract for total amphiphilic (TAC) and lipophilic compounds (TLC).

In Figure 7 and Figure 8, the total phenolic content (TPC), expressed as mg gallic acid equivalents per gram of extract (mg GAE/g extract), demonstrated marked differences between lipid fractions (TLC vs. TAC), product type (beverage vs. yogurt), and botanical origin (soy vs. oat).

In soy-based products, TLC extracts of yogurt exhibited significantly higher phenolic content compared with the corresponding TAC extracts. Specifically, soy yogurt TLC presented a mean TPC of 21.74 ± 3.00 mg GAE/g extract, whereas the respective TAC fraction contained 2.02 ± 0.33 mg GAE/g extract. Furthermore, a comparison between soy beverage and soy yogurt revealed that fermentation increased the phenolic content in the TLC fraction. The soy beverage TLC showed a mean TPC of 6.43 ± 0.71 mg GAE/g extract, substantially lower than that observed in soy yogurt TLC (21.74 ± 3.00 mg GAE/g extract).

Overall, these findings indicate that in soy-based yogurt, phenolic compounds are predominantly associated with the lipophilic fraction and that fermentation enhances their extractable concentration. This increase may be attributed to microbial enzymatic activity during fermentation, leading to the release of bound phenolics or biotransformation into more extractable forms.

In oat-based preparations, distinct patterns were observed. In oat beverages, TLC extracts contained significantly higher phenolic levels (32.55 ± 2.72 mg GAE/g extract) compared with the corresponding TAC extracts (6.65 ± 5.10 mg GAE/g extract). However, in contrast to soy, fermentation of oat products resulted in a reduction in phenolic content within the TLC fraction. Specifically, oat yogurt TLC extracts exhibited a mean TPC of 7.15 ± 1.19 mg GAE/g extract, markedly lower than that of oat beverage TLC extracts (32.55 ± 2.72 mg GAE/g extract).

Comparative analysis between botanical origins further revealed statistically significant differences. In beverage samples, oat TLC extracts contained substantially higher TPC (32.55 ± 2.72 mg GAE/g extract) than soy TLC extracts (6.43 ± 0.71 mg GAE/g extract). Conversely, in yogurt-type products, soy TLC extracts (21.74 ± 3.00 mg GAE/g extract) demonstrated higher phenolic content than oat TLC extracts (7.15 ± 1.19 mg GAE/g extract).

Collectively, these results highlight that phenolic distribution is strongly influenced by both the extraction fraction and the fermentation process, with distinct responses depending on plant origin. While oat beverages are characterized by particularly high phenolic content in the lipophilic fraction, fermentation appears to enhance phenolic availability in soy products but reduce it in oat-based formulations. These differences may reflect variations in matrix composition, phenolic binding forms, and microbial metabolic pathways during fermentation. Moreover, other non-phenolic substances from food, including organic acids, are also reduced by the F–C reagent [37], thus leading to overestimation of the phenolic content, probably at the TLC fractions where organic fatty acids are also migrated.

Finally, a wide range of TPC in soy-derived drinks has also previously been reported, varying from 30 to 320 mg GAE/100 g of drink, depending on the soybean variety and the processing conditions to produce soymilk [38,39], which, if translated to mg GAE/g of extract from 30 mL of drink that was performed in the present study compared to 100 mL drink studied in these previous studies (approximately 10 to 100 mg of GAE/g of extract derived from 30 mL drink), shows that there is a consistency in the phenolics content of the soy beverages with previously reported outcomes. In contrast, in the present study, much higher amounts of oat-derived total phenolic content were identified in the extract of the oat beverage in comparison to previous studies for other oat-derived beverages (approx. 0.310 mg GAE/g dry matter of the beverage) [40]. Similarly, both soy and oat-derived yogurt-style products of the present study and their TAC and TLC extracts were found to contain higher total phenolic content than the previously reported ones for such products (approx. 3–5 mg GAE/g for soy yogurt [41] and 0.4–0.6 mg GAE per L of yogurt [42]). This may be an indication that within the extracts and after the evaporation process of the samples, the total phenolic content in both TAC and TLC extracts is concentrated in comparison to the 100 mL of the beverage, the 1 g, or the 1 L of the yogurts previously studied.

**Figure 9:**
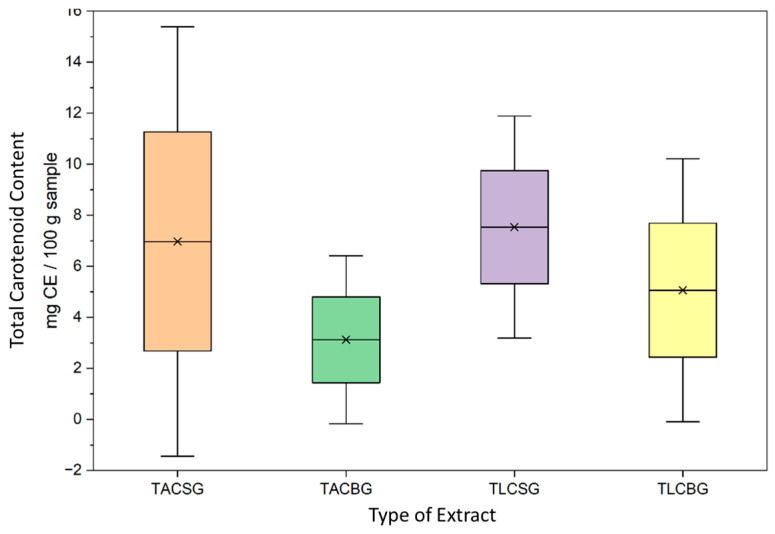
Comparison of total carotenoid content in soy and oat beverages, expressed as mg carotenoid equivalent/g extract for total amphiphilic (TAC) and lipophilic compounds (TLC).

**Figure 10:**
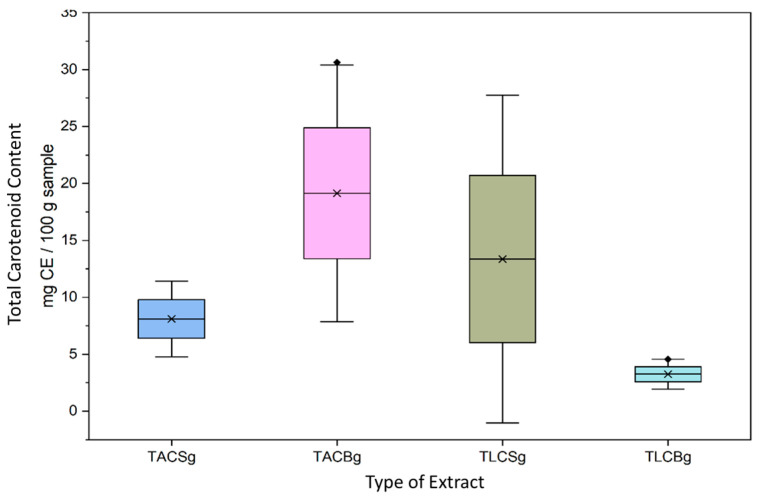
Comparison of total carotenoid content in soy and oat yogurts, expressed as mg carotenoid equivalent/g extract for total amphiphilic (TAC) and lipophilic compounds (TLC).

With respect to the carotenoid content of all extracts, a statistically significant difference was observed between the total amphiphilic compound (TAC) extracts of oat beverage and oat yogurt in Figure 9 and Figure 10. Specifically, oat yogurt exhibited a markedly higher total carotenoid content (19.14 ± 9.97 mg β-carotene equivalents (CE)/g extract in TAC and 3.25 ± 1.16 mg CE/g of TLC extract) compared with the corresponding oat beverage (approx. 3 mg CE/g TAC extract-5 mg CE/g TLC extract), and especially in the TAC extracts of these oat-derived products. These findings indicate that fermentation substantially enhances carotenoid extractability in oat-based TAC matrices.

Furthermore, within oat yogurt samples, TAC extracts contained significantly higher carotenoid concentrations than the total lipophilic compound (TLC) extracts (19.14 ± 9.97 vs. 3.25 ± 1.16 mg CE/g extract, respectively), highlighting the preferential association of carotenoids with amphiphilic lipid fractions.

In plant-based beverages, total carotenoid content varied significantly according to both botanical origin (soy vs. oat) and lipid fraction (TL, TAC, TLC). Across all beverages, TAC fractions consistently exhibited the highest carotenoid concentrations, corroborating previous reports that carotenoids are predominantly localized within polar and amphiphilic lipid environments. In contrast, TLC fractions demonstrated comparatively lower values, likely due to the limited solubility and stabilization of carotenoids in strictly nonpolar lipid matrices.

Soy beverages generally exhibited higher carotenoid concentrations than oat beverages, a finding consistent with literature suggesting enhanced incorporation and bioavailability of fat-soluble micronutrients in soy-based systems. Overall, both soy- and oat-based beverages may be considered dietary sources of carotenoids contributing to antioxidant activity, with soy demonstrating a moderately stronger carotenoid profile.

In yogurt-type products, carotenoid concentrations were consistently higher than in the corresponding non-fermented beverages. This enhancement is attributed to the fermentation process, during which microbial activity—primarily by *Lactobacillus* spp. and *S. thermophilus*—facilitates matrix degradation, disrupts cell wall structures, and promotes the release of bound carotenoids and phenolic compounds. Such biochemical transformations may improve carotenoid bioaccessibility and stability.

In soy yogurt samples, TAC fractions exhibited significantly higher carotenoid concentrations than TLC fractions (*p* < 0.05), supporting evidence that carotenoids are preferentially stabilized within amphiphilic systems. A similar distribution pattern (TAC > TL > TLC) was observed in oat yogurts, although absolute concentrations were slightly lower. This pattern may reflect the contribution of polar oat constituents, such as β-glucans, which can enhance carotenoid retention through physicochemical interactions within amphiphilic matrices.

Collectively, these results suggest that fermented plant-based products—particularly soy yogurt-type formulations—represent an important source of carotenoids with potentially enhanced antioxidant functionality.

Interestingly, even though the phenolic compounds of plant-derived sources are usually thought to be the relatively more polar compounds from all the other amphiphilic compounds of such sources, they are usually expected to be found in much higher amounts in the TAC fractions of the plant derived extracts, where they mainly migrate during the counter-current distribution as more efficiently dissolved in the hydroalcoholic solvent of this process. The opposite is usually observed for the carotenoids, which are less polar amphiphilic compounds than the phenolics, and thus it is usually more difficult to separate them from the TLC fractions towards the TAC fractions, where they indeed belong to a molecular species of compounds. Thus, during the counter-current distribution, a partition of both carotenoids and phenolics is usually observed between TAC and TLC, with TAC favoring for phenolics and polar lipid amphiphilic compounds and some carotenoids (mostly those of low molecular weight), while TLC favoring for the more lipophilic compounds including triglycerides, phytosterols, cholesterol esters and some carotenoids of high molecular weights that are usually trapped in these lipophilic conditions.

Nevertheless, this was not the case in the present study, and it seems that more complex mechanisms exist for such separations that might also be dependent on the plant-based matrices being separated. For example, in the present study, the TLC fractions were rich in phenolic compounds. This may arise from two complementary factors. First, the newly used ethanol/hexane counter-current distribution system used in the modified protocol seems not to separate the phenolics from the lipophilic content of the whole extract as efficiently as previously observed when petroleum ether (a more lipophilic solvent) was used for such separations. Alternatively, or simultaneously, it is highly possible that esterified or lipid-associated phenolic compounds, often present in plant matrices, are also present in our samples. These can be phenolic esters or phenolic–lipid conjugates, which exhibit increased lipophilicity and therefore partition preferentially into the nonpolar, more lipophilic fractions like the TLC ones of the present study.

In contrast, carotenoids—although highly hydrophobic—may associate with amphiphilic lipid environments such as polar lipid-rich fractions, which explains their higher prevalence in the TAC fractions. The amphiphilic nature of polar lipids can stabilize carotenoids through hydrophobic interactions within lipid bilayer-like structures, enhancing their recovery in these fractions.

Moreover, the copresence of all these types of bioactives (phenolics, carotenoids, and polar lipids) in these extracts contributes synergistically to the overall health-promoting benefits associated with soy and pat-derived plant-based dairy alternatives. More specifically, the antioxidant efficiency of these extracts can be substantially enhanced through synergistic interactions between phenolics, carotenoids, and amphiphilic lipid components, enabling protection across both aqueous and lipid phases. Phenolic compounds primarily act as potent radical scavengers and modulators of redox signaling, directly neutralizing reactive oxygen species (ROS) and preventing oxidative damage to biomolecules, including membrane lipids, while also activating endogenous antioxidant pathways (e.g., through the Nrf2 translation factor). In parallel, the more lipophilic constituents than phenolics, meaning the carotenoids and polar lipids (rich in unsaturated fatty acids), localize within hydrophobic domains like biological membranes, where they inhibit lipid peroxidation and stabilize membrane structure. The amphiphilic nature of polar lipids facilitates the formation of interfacial systems (e.g., emulsions or membrane bilayers) that spatially co-localize hydrophilic phenolics and lipophilic antioxidants, thereby promoting complementary and possibly regenerative antioxidant interactions at the water–lipid interface. This cooperative arrangement enhances radical scavenging efficiency by enabling sequential electron or hydrogen transfer mechanisms across phases, improving the overall redox balance and providing more effective protection against oxidative and inflammatory damage in complex biological environments such as cell membranes and other formations where polar lipids are bio-functional structural elements, like human plasma lipoproteins, including low-density lipoprotein (LDL).

Oxidation of low-density lipoprotein (LDL) is widely recognized as a key factor in the development of atherosclerosis, suggesting that increased resistance of LDL to oxidative modification may help prevent atherogenesis and related disorders [3]. Various plant-derived secondary metabolites have been investigated for their protective effects against LDL oxidation, with many phenolic compounds and carotenoids shown to enhance its oxidative stability. On their own, some phenolics like the flavonoid glycoside rutin effectively inhibited copper-induced formation of conjugated dienes and the loss of tryptophan fluorescence in LDL, while enrichment of LDL with some carotenoids on their own (i.e., lutein or lycopene alone) did not significantly reduce oxidation [43]. On the other hand, combinations of rutin with either lutein or lycopene demonstrated synergistic, supra-additive protection against LDL oxidation, likely due to their differential interactions with the polar lipids of LDL and thus their distinct localization within the LDL particle, as well as due to their complementary mechanisms of antioxidant action [43]. Taking into account similar outcomes from several other studies, it is profound that the copresence of all these bioactive amphiphilic molecules in both soy- and oat-derived dairy alternative products can provide supra-additive synergistic activities against several thrombo-inflammatory and oxidative stress-related complications [3,4].

### 3.4. Determination of Total Antioxidant Activity

**Figure 11:**
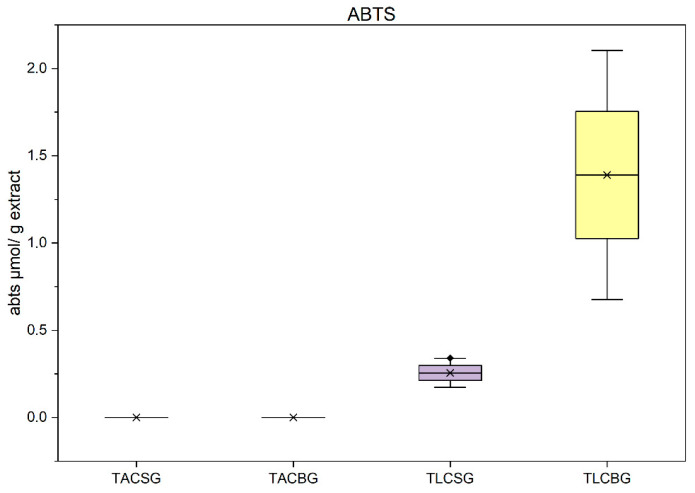
Quantification of the antioxidant capacity of soy and oat beverages using the ABTS method, expressed as mmol TE/g extract for total amphiphilic (TAC) and lipophilic compounds (TLC).

**Figure 12:**
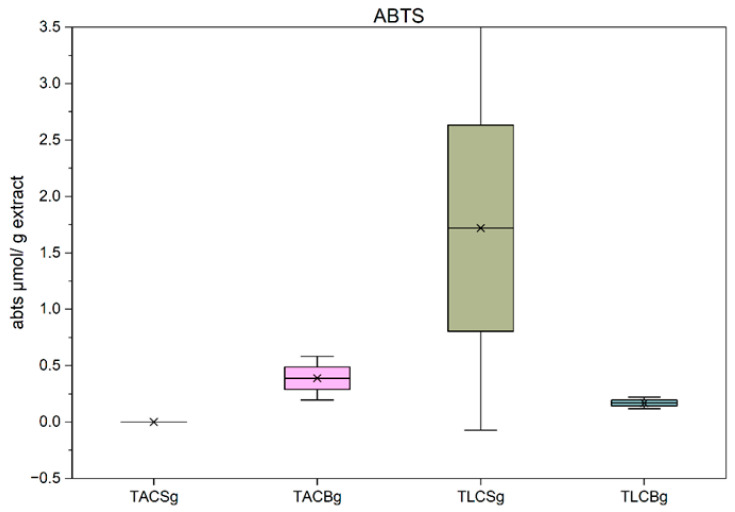
Quantification of the antioxidant capacity of soy and oat yogurts using the ABTS method, expressed as mmol TE/g extract for total amphiphilic (TAC) and lipophilic compounds (TLC).

**Figure 13:**
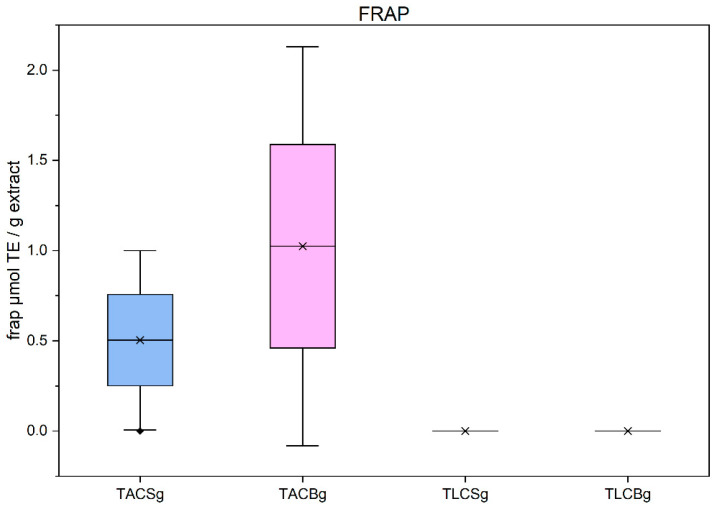
Quantification of the antioxidant capacity of soy and oat yogurts using the FRAP method, expressed as mmol TE/g extract for total amphiphilic (TAC) and lipophilic compounds (TLC).

**Figure 14:**
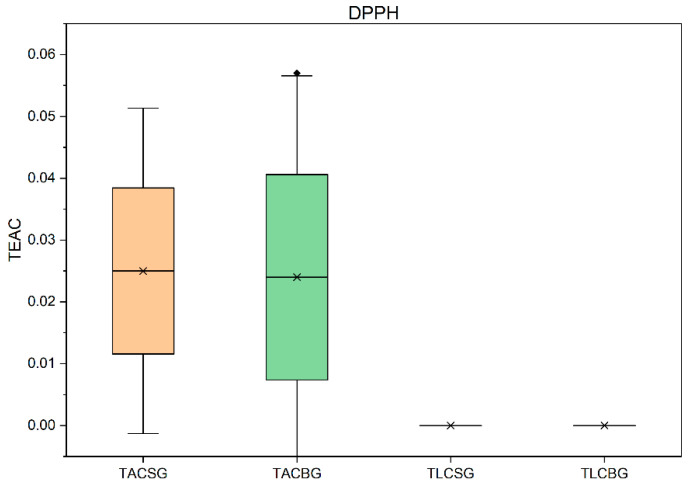
Quantification of the antioxidant capacity of soy and oat beverages using the DPPH method, expressed as TEAC for total amphiphilic (TAC) and lipophilic compounds (TLC).

The antioxidant activities of all extracts were evaluated using three different and distinct bioassays: DPPH, ABTS, and FRAP. The results of these analyses are summarized in Figure 11, Figure 12, Figure 13 and Figure 14. More specifically, FRAP measures the ferric-reducing antioxidant activity of the antioxidant bioactives that are present in each extract, while both DPPH and ABTS assays measure free radical scavenging antioxidant activity of antioxidant bioactives present in an extract; still, there are differences between these two methodologies.

The ABTS assay measures antioxidant capacity primarily through a single electron transfer (SET) mechanism, where antioxidants reduce the pre-formed blue/green ABTS•+ radical cation back to its colorless neutral form. The reduction is monitored spectrophotometrically at 734 nm, providing a quantitative measure of electron-donating ability compared to a well-established antioxidant molecule, Trolox, which is a synthetic analog of vitamin E, with much more hydrophilic properties than vitamin E. Thus, by ABTS, the total antioxidant capacity for all compounds present in a bioactive extract can be determined as the antioxidant capacity of both amphiphilic (relatively more polar compounds) and lipophilic (less polar) compounds being present and can scavenge free radicals by such a mechanism, while other assays that use different mechanisms usually favor the detection of antioxidant activities of the more polar amphiphilic compounds, like in the case of the DPPH assay. This is why such assays are used in combination, meaning to fully evaluate if the free radical scavenging activity present takes place mostly from the amphiphilic or from the lipophilic components of the extract. Overall, an electron transfer probe in antioxidant assays like the ABTS one is often used alongside hydrogen atom transfer (HAT)-based assays (like DPPH or ORAC) to distinguish between antioxidants that act via hydrogen donation (favoring polar amphiphilic compounds with hydroxyl functional groups like the phenolic compounds) versus electron transfer (favoring both amphiphilic and lipophilic compounds capable of this mechanism).

Unlike SET, HAT is a fundamental radical reaction involving the concerted transfer of a proton and an electron (H^+^ + e^−^) in a single kinetic step, often utilized to generate reactive alkyl radicals from non-activated C–H bonds. HAT is a primary mechanism by which antioxidants bearing hydroxyl functional groups in their structures (ROH) neutralize the 2,2-diphenyl-1-picrylhydrazyl (DPPH•) radical; the antioxidant transfers a hydrogen atom to DPPH• to form a non-radical reduced species, DPPH-H, reducing the purple color of the radical. This process depends heavily on the bond dissociation enthalpy (BDE) of the antioxidant. HAT efficiency is dictated by the antioxidant’s structure, solvent polarity, and the stability of the resulting radical. Nevertheless, the DPPH assay measures antioxidant capacity by reactions involving mixed mechanisms (HAT/SET) depending on concentration. Thus, apart from HAT, the DPPH reaction is also driven by the SET mechanism, where an antioxidant (AOH) transfers an electron to the purple DPPH radical, reducing it to yellow DPPH-H. The reduction in this mixed model is monitored spectrophotometrically at 519 nm, providing a quantitative measure of both HAT (mainly) and SET (less) ability, often expressed as Trolox Equivalent Antioxidant Capacity (TEAC), when compared to that of Trolox. While often occurring alongside HAT, SET-based DPPH reduction is highly influenced by the solvent polarity and the ionization potential of the antioxidant. Also, while DPPH and some other assays run through such a mixed model, HAT is still the dominant process for phenolic antioxidants in many environments and in this assay.

#### 3.4.1. Evaluation Using ABTS Method

The assessment of antioxidant capacity using the ABTS radical cation decolorization assay revealed pronounced differences between soy- and oat-derived extracts. As illustrated in Figure 11, the lipophilic fractions (TLC) of the oat beverage exhibited the highest antioxidant activity, followed by the corresponding TLC fraction of the soy beverage. In contrast, the total amphiphilic compound (TAC) fractions of both plant sources demonstrated negligible activity under the conditions of this assay. These findings indicate that, in non-fermented beverages, antioxidant capacity as measured by ABTS is primarily attributable to constituents present or trapped in the lipophilic conditions of the TLC fractions.

This observation is noteworthy, as previous studies frequently report higher antioxidant activity in soy products, largely attributed to their isoflavone and phenolic content. However, in the present samples, the superior performance of oat TLC fractions may be associated with the higher phenolic content observed in these fractions, unlike other studies where the phenolics were present mainly in the TAC fractions; the antioxidant activity was better in these fractions, too. Thus, the antioxidant activity observed in soy TLC fractions can be attributed to the presence of tocopherols and other lipid-soluble antioxidant molecules. The limited activity of TAC fractions in both plant matrices may reflect either lower concentrations of ABTS-reactive compounds or reduced stability and reactivity of polar antioxidants under the specific assay conditions, or less efficient separation of organic molecules from the TLC towards the TAC fraction in the conditions applied during the counter-current distribution process.

In yogurt-type products (Figure 13), a different pattern emerged compared with the corresponding beverages. The TLC fraction of soy yogurt exhibited the highest antioxidant capacity overall. Notably, the TAC fraction of oat yogurt demonstrated substantially greater activity than the TAC fraction of the oat beverage. This enhancement is likely associated with the fermentation process, which has been shown to increase the bioavailability of phenolic constituents—such as avenanthramides and hydroxycinnamic acids—through enzymatic hydrolysis and matrix degradation. Microbial enzymes, including β-glycosidases and esterases, may facilitate the release of bound phenolics from the TLC towards the TAC fractions, thereby enhancing their measurable antioxidant activity.

Collectively, these results suggest that whereas lipophilic fractions dominate the ABTS response in beverages, fermentation promotes a relative increase in the contribution of amphiphilic fractions in yogurt-type products, particularly in oat-based matrices. This trend aligns with literature indicating that fermentation can enhance antioxidant capacity through improved phenolic bioavailability and the generation of novel bioactive metabolites.

#### 3.4.2. Evaluation Using FRAP Method

Antioxidant capacity evaluated by the ferric reducing antioxidant power (FRAP) assay demonstrated a distinct profile compared with the ABTS results. Measurable reducing activity was detected exclusively in fermented yogurt-type products, whereas both soy and oat beverages exhibited negligible activity (Figure 13).

Among the analyzed samples, the TAC fraction of oat yogurt displayed the highest FRAP value, significantly exceeding that of soy yogurt TAC. In contrast, the TLC fractions of both soy and oat products showed minimal or no detectable reducing capacity, indicating that lipophilic constituents contribute negligibly to antioxidant activity as measured by this electron-transfer-based assay.

The exclusive detection of FRAP activity in yogurt samples suggests that fermentation promotes the formation or release of compounds with enhanced ferric ion-reducing potential. These compounds are likely polar or amphiphilic in nature, consistent with the enrichment of bioaccessible phenolics following microbial enzymatic action. Overall, the FRAP results reinforce the conclusion that fermentation enhances the reducing power of plant-based dairy alternatives, primarily through modifications of the amphiphilic bioactive fraction.

#### 3.4.3. Evaluation Using DPPH Method

The determination of antioxidant capacity using the DPPH radical scavenging assay (Figure 14) in soy- and oat-based beverages demonstrated that antioxidant activity is predominantly associated with the total amphiphilic compound (TAC) fractions. Both soy TAC and oat TAC extracts exhibited comparable radical scavenging activity, although slightly greater variability was observed among oat samples. In contrast, the total lipophilic compound (TLC) fractions showed negligible DPPH• neutralization capacity, indicating that lipophilic constituents do not substantially contribute to antioxidant activity as measured by this assay.

The DPPH method is based on the ability of antioxidant molecules to donate a hydrogen atom to stabilize the DPPH radical, thereby reducing its absorbance. Consequently, compounds with effective hydrogen-donating capacity—particularly polar phenolics—are preferentially detected. The present findings are consistent with previous reports demonstrating that phenolic compounds in soy (e.g., isoflavones) and oats (e.g., avenanthramides and ferulic acid) are major contributors to radical scavenging activity. The greater dispersion observed in oat samples may reflect differences in phenolic composition, concentration, and distribution, as previously described in the literature.

Overall, these results indicate that, in the DPPH assay, TAC fractions serve as the principal contributors to antioxidant capacity in both soy and oat beverages, with comparable overall activity between the two plant sources. This pattern contrasts with the ABTS assay results, in which TLC fractions predominated, underscoring the influence of assay mechanism on the detected antioxidant profile. Specifically, as aforementioned, DPPH favors hydrogen atom-donating polar antioxidants of more polar compounds like phenolics, whereas ABTS may respond more broadly to single electron transfer of both amphiphilic and lipophilic radical scavenging components.

Notably, antioxidant activity was also detected in the TAC extracts of soy yogurt, with an average Trolox Equivalent Antioxidant Capacity (TEAC) value of approximately 0.019. This magnitude of activity is comparable to that observed in the corresponding beverages, suggesting that fermentation maintains or modestly enhances amphiphilic radical scavenging capacity in soy-based yogurt-type products.

### 3.5. Fatty Acid Composition of the PL Bioactives Present in the TAC Extracts from Dairy Alternatives Based on Soy and Oat

The fatty acid composition of the PL in the TAC extracts derived from soy and oat dairy alternatives was determined by liquid chromatography–mass spectrometry (LC–MS), and the data are demonstrated in Table 2. Particular emphasis was placed on samples analyzed after saponification, as this procedure enables the identification of fatty acids esterified to polar lipid fractions, thereby providing insight into the fatty acid profile associated with amphiphilic PL bioactive components. In contrast, Table 3 shows the fatty acid composition of the free fatty acids of the TAC extracts, as saponification of the samples did not occur prior to the determination and quantification. The results are expressed as the relative percentage contribution of each fatty acid within the TAC extracts.

LC–MS analysis demonstrated that the saturated fatty acids (SFA) were the main fatty acids of the PL in the TAC extracts of all products, with soy beverage PL containing approx. 90% SFA, and PL from oat beverage 86% SFA, while the PL from their fermented yogurt-type products contained higher amounts of SFA (approx. 95% each). Stearic acid (C18:0) was the predominant fatty acid in all samples, with values ranging from 40.8% in oat beverages to 48.2% in the PL from soy yogurt. Palmitic acid (C16:0) was also present at consistently high levels (34.0–36.2%), while lauric acid (C12:0) and myristic acid (C14:0) were detected at lower concentrations in all PL (4.3–5.6% and 1.7–2.1%, respectively). These findings indicate that saturated fatty acids (SFAs) constitute the dominant lipid class of the PL in the TAC extracts of both soy and oat products, in agreement with previous reports on soy and other plant-based dairy alternatives [14,36].

Interestingly, differences were observed between the unsaturated fatty acid (UFA), monounsaturated fatty acid (MUFA), and polyunsaturated fatty acid (PUFA) contents of the PL from the non-fermented soy and oat beverages and those of the PL from the fermented yogurt-type products. For example, the amount of UFA, MUFA, and PUFA in the PL of TAC was decreased after fermentation, but the type of PUFA and MUFA that were reduced were different in each case. More specifically, UFA of the PL from soy drink reached approx. 9%, constituted by 5% from PUFA and 4% of MUFA with the main representative ones being the omega-6 (*n*-6) PUFA linoleic acid (*n*-6 C18:2) of 4.75%, followed by the omega-9 (*n*-9) MUFA oleic acid (*n*-9 C18:1) of 3.5%, and the alpha linolenic acid (*n*-3 18:3) from the omega-3 (*n*-3) PUFA, ranging between 0.6 and 0.7%, followed by traces of eicosapentaenoic acid (EPA: *n*-3 C20:5). Therefore, the *n*-6/*n*-3 PUFA ratio for the PL of the TAC from the soy drink reached values of 7.5. When this ratio is higher than 15–20/1 in a food source, *n*-6 PUFA predominates; since the *n*-6 PUFA are pro-inflammatory lipids from which eicosanoids are usually produced, a value higher than 20/1 for the *n*-6/*n*-3 PUFA ratio indicates that the specific food is characterized as a pro-inflammatory, similarly to the processed foods in Western and Westernized diets [44]. On the other hand, the lower the value for this ratio (as close to the value 1 as possible), the better the anti-inflammatory profile for the food source and the extract containing such low levels of this ratio [44]. For the soy drink, this ratio reached levels of 7–8%, which is lower than the 15/1 threshold, indicating an anti-inflammatory profile for the PL of the soy drink.

Similarly, UFAs of PLs from oat drink consisted of higher MUFA content (approx. 13%) than observed in soy PLs, with similar PUFA content (5%); main representatives were *n*-9 MUFA oleic acid (*n*-9 C18:1, 8.7%), followed by *n*-6 PUFA linoleic acid (*n*-6 C18:2, 4.75%) and alpha-linolenic acid (*n*-3 C18:3, 0.2–0.3%), followed by traces of eicosapentaenoic acid (EPA; *n*-3 C20:5). Therefore, the *n*-6/*n*-3 PUFA ratio for the PL of the TAC from the oat drink reached values of 17–18/1, which are close to the values for the pro-inflammatory Westernized foods.

In contrast, in the PL of the TAC from the fermented soy and oat yogurt-type product, their UFA were reduced to approx. 3%, constituted by a reduction in PUFA that reached up to 0.8% and 2% for the MUFA with the main representative ones being again the *n*-6 PUFA linoleic acid (*n*-6 C18:2), but in much lower amounts this time, reaching levels of 0.55%, followed by the *n*-9 MUFA oleic acid (*n*-9 C18:1) of 2%, and again the alpha linolenic acid (*n*-3 18:3) from the *n*-3 PUFA ranging between 0.2 and 0.3%, followed by traces of eicosapentaenoic acid (EPA: *n*-3 C20:5). The reduction in the *n*-6 PUFA and the retaining of the *n*-3 PUFA after fermentation resulted also in a major reduction in the levels of the *n*-6/*n*-3 PUFA ratio for these PL of both the fermented products, which reached values very close to one (2.24 and 2.82, respectively), suggesting that both the fermented products from oat and soy contain PL with favorable anti-inflammatory levels of this ratio that makes these foods bio-functional against inflammation, thrombosis and associated chronic disorders, including cardiovascular diseases and cancer. This result comes in accordance with previous ones reported for other plant-based fermented yogurt-type dairy alternative products, such as those from rice and almond, and to a lesser extent from coconut.

With respect to the unsaponified free fatty acids that were detected in the TAC extracts of all samples due to partition during extraction, the obtained data presented in Table 3 shows similar trends occurring for the fatty acids detected in the fermented versus the fatty acids of the unfermented products, with much higher UFA, PUFA and especially *n*-3 PUFA content being detected in the fermented products, providing them with much lower *n*-6/*n*-3 PUFA values and thus supporting their anti-inflammatory functional health-promoting properties.

### 3.6. Evaluation of Anti-Inflammatory and Anti-Platelet Properties of Soy and Oat Extracts

In order to evaluate the anti-inflammatory and antithrombotic potential of the TAC extracts from all products the platelet aggregometry bioassay was utilized, as it is a precise, accurate, simple, fast and replicable methodology to define the anti-inflammatory and antiplatelet potential of each sample by determining their inhibitory effects on platelet aggregation mediated through two distinct activation pathways: the inflammatory platelet-activating factor (PAF) pathway and the thrombotic adenosine diphosphate (ADP) pathway. Inhibitory potency was expressed as IC_50_ values (μg extract), defined as the concentration required to inhibit 50% of maximum reversible platelet aggregation. Lower IC_50_ values indicate greater biological activity. The analysis focused exclusively on the total amphiphilic compound (TAC) fractions obtained from both beverage and yogurt-type products.

#### 3.6.1. Inhibitory Activity via the PAF Pathway

**Figure 15:**
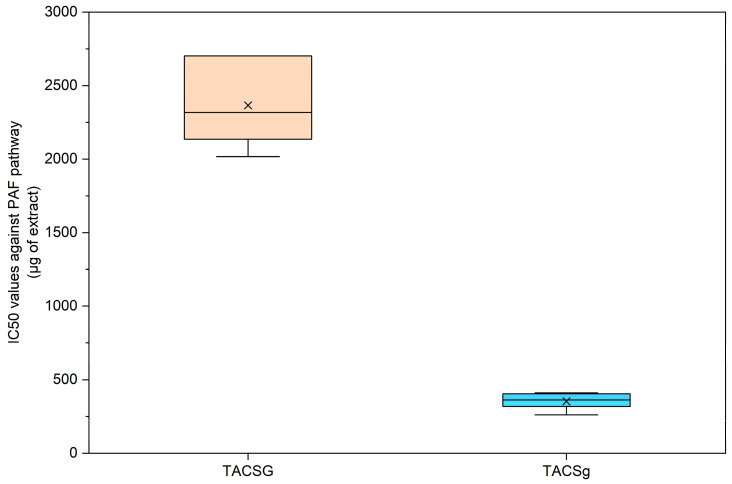
Inhibitory effects of total amphiphilic compounds (TACs) from soybean products against the PAF pathway. Results are expressed as IC_50_ values (half-maximal inhibitory concentration), meaning the mass of the compound extract in μg present in the aggregometer cuvette containing 250 μL of human platelet-rich plasma (hPRP) that can cause 50% of inhibition of the PAF-induced inflammatory activation and aggregation of hPRP (the lower the IC_50_ value, the more potent the anti-inflammatory activity for an extract).

**Figure 16:**
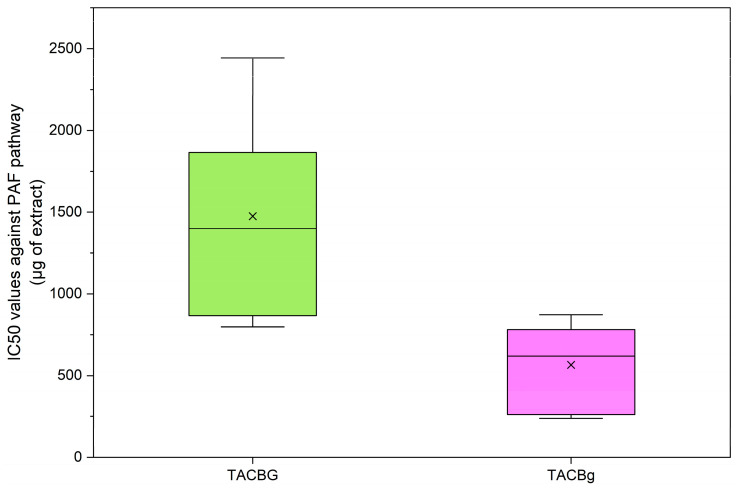
Inhibitory effects of total amphiphilic compounds (TACs) from oat products against the PAF pathway. Results are expressed as IC_50_ values (half-maximal inhibitory concentration), meaning the mass of the compound extract in μg present in the aggregometer cuvette containing 250 μL of human platelet-rich plasma (hPRP) that can cause 50% of inhibition of the PAF-induced inflammatory activation and aggregation of hPRP (the lower the IC_50_ value, the more potent the anti-inflammatory activity for an extract).

In rice-, almond-, and coconut-derived beverages (non-fermented dairy alternative drinks) and their fermented products, a huge difference has previously been reported between the bioactivities of their TAC extracts and those of their TLC extracts against PAF-induced or ADP-induced aggregation of human platelets [14]. Similarly, in the present study, the TAC extracts of all oat and soy-derived fermented and non-fermented samples showed much stronger anti-inflammatory properties against PAF and antiplatelet effects against ADP. Thus, Figure 15 and Figure 16 show the potent bioactivities of the TAC extracts from these sources against these two thrombo-inflammatory mediators, respectively.

More specifically, the TAC extract derived from soy beverage exhibited the highest IC_50_ value (2365.05 μg), indicating relatively weak antiplatelet activity against PAF-mediated activation. In contrast, the TAC extract from soy yogurt demonstrated a substantially lower IC_50_ value (352.83 μg), reflecting a markedly enhanced inhibitory effect. Notably, this value was the lowest among all analyzed samples, indicating that soy yogurt possessed the strongest anti-PAF activity, which was also found to be comparable to that from almond and rice and better than that from coconut [14].

The pronounced reduction in IC_50_ following fermentation suggests that microbial processing significantly enhances the antiplatelet capacity of soy-based products by altering their polar lipid profile, something that has previously been observed not only on plant-based dairy alternatives [14], but also in dairy fermented products (yogurt, yogurt drink, and cheese) versus non-fermented ones (milk) [14,45,46,47]. This enhancement may be attributed to the release, structural modification, or increased bioavailability of amphiphilic bioactive compounds during fermentation, thereby improving their ability to interfere with PAF-mediated platelet activation pathways.

In oat-derived samples (TACBG and TACBg), the yogurt-type product (TACBg) exhibited significantly lower IC_50_ values compared with the corresponding beverage (TACBG), indicating a markedly enhanced inhibitory effect on PAF-induced platelet aggregation following fermentation.

Additionally, the reduced variability observed in TACBg suggests a more consistent and stable bioactive profile relative to the non-fermented beverage. The improvement in antiplatelet activity after fermentation may be attributed to the increased bioavailability or release of lipid-soluble bioactive compounds, including tocopherols and polyunsaturated fatty acids, which have been previously reported to exert inhibitory effects on the PAF signaling pathway [44].

#### 3.6.2. Inhibitory Action Through the ADP Pathway

**Figure 17:**
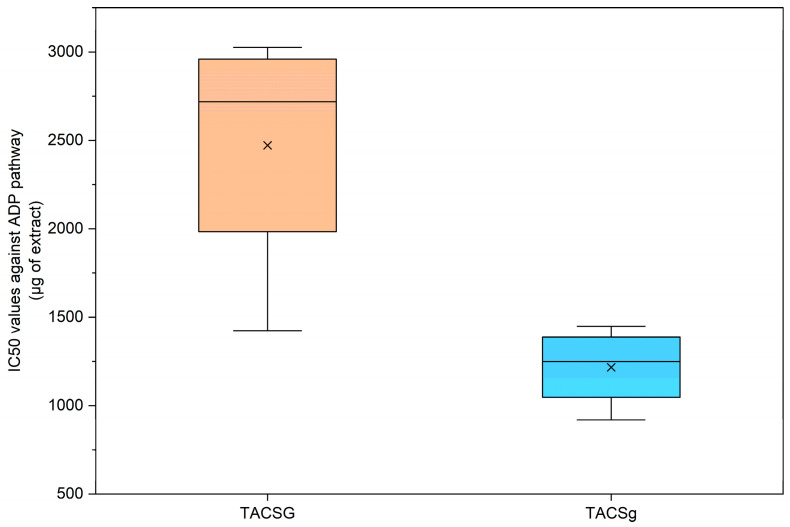
Anti-platelet effects of total amphiphilic compounds (TACs) from soy products against the classic platelet agonist ADP. Results are expressed as IC_50_ values (half-maximal inhibitory concentration), meaning the mass of the compound extract in μg present in the aggregometer cuvette containing 250 μL of human platelet-rich plasma (hPRP) that can cause 50% of inhibition of the ADP-induced thrombotic activation and aggregation of hPRP (the lower the IC_50_ value, the more potent the anti-inflammatory activity for an extract).

Regarding ADP-induced platelet aggregation, soybean-derived samples exhibited a pattern comparable to that observed for the PAF pathway. Specifically, the TAC extract from soy beverage demonstrated the highest IC_50_ value (2471.8 μg), indicating relatively weak antiplatelet activity (Figure 17). In contrast, the TAC extract from soy yogurt displayed a substantially lower IC_50_ value (1220.3 μg), reflecting enhanced inhibitory capacity following fermentation.

This improvement is consistent with previous evidence suggesting that soybean fermentation promotes the enzymatic hydrolysis of isoflavone glycosides and storage proteins into smaller, more bioactive molecules, including isoflavone aglycones and bioactive peptides. These compounds may exhibit greater affinity or efficacy in modulating platelet activation mediated through the ADP signaling pathway.

Moreover, the limited variability observed in the TAC extract of soy yogurt (TACSg) indicates a stable and homogeneous inhibitory effect, further supporting the role of fermentation in enhancing both the potency and consistency of antiplatelet activity.

In contrast to the findings observed in soy-derived samples, the oat beverage (TACBG) demonstrated lower IC_50_ values than the corresponding oat yogurt (TACBg), indicating stronger inhibitory activity against ADP-induced platelet aggregation in the non-fermented product.

**Figure 18:**
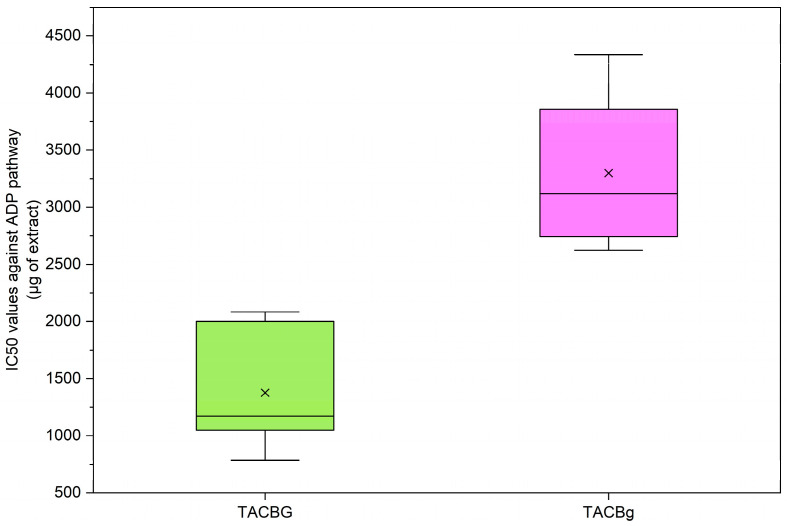
Anti-platelet effects of total amphiphilic compounds (TACs) from oat products against the classic platelet agonist ADP. Results are expressed as IC_50_ values (half-maximal inhibitory concentration), meaning the mass of the compound extract in μg present in the aggregometer cuvette containing 250 μL of human platelet-rich plasma (hPRP) that can cause 50% of inhibition of the ADP-induced thrombotic activation and aggregation of hPRP (the lower the IC_50_ value, the more potent the anti-inflammatory activity for an extract).

The increase in IC_50_ values following fermentation suggests that microbial processing may attenuate the activity of specific oat-derived bioactive compounds involved in the modulation of the ADP pathway (Figure 18). This reduction in potency may be associated with structural modifications of phenolic constituents or alterations in β-glucan configuration and interactions during fermentation, potentially affecting their biological efficacy.

Nevertheless, the TAC extract of oat yogurt (TACBg) retained measurable antiplatelet activity, indicating that fermentation does not eliminate bioactive components entirely but rather modulates their relative abundance, structure, or functional properties.

### 3.7. Structural Elucidation and Proposed Structures of the Main Bioactive Phospholipids Detected in the TAC Extracts of Both the Oat and Soy Products

Liquid chromatography–mass spectrometry (LC–MS) analysis generated distinct chromatographic profiles for all four matrices (soy beverage, soy yogurt-type product, oat beverage, and oat yogurt-type product), enabling detailed characterization of their fatty acid and polar lipid composition and confirming the presence of structurally diverse polar lipids in both fermented and non-fermented samples.

In all products, polar lipids were predominantly represented by phosphatidylcholines (PCs) and phosphatidylethanolamines (PEs), comprising multiple fatty acid combinations, as previously described for soy [36], but also for other plant-based dairy alternatives [14]. Nevertheless, it is worth mentioning that, as shown in Table 4 and Table 5, several acyl-alkyl polar lipids were detected in both the fermented and the unfermented products from both plant-derived sources (oat and soy), which have previously been characterized as PAF-analogs and thus they have previously presented a strong anti-PAF agonistic effect due to their structural homology with the PAF-receptor, with the structure of the classic molecule of PAF (1-*O*-alkyl-16:0-2-*sn*-acetyl-glycerol phosphatidylcholine) [3,4,32]. The presence of many of these ether-ester (acyl-alkyl) glycerol-based polar lipids with antagonistic effects against PAF seems to explain, through a structure–activity relationship, the aforementioned potent anti-PAF effects of all samples tested in human platelets.

In addition, the fatty acids present in the *sn*-2 position of the glycerol backbone of these polar lipids seems also to play an important role for both their anti-PAF and anti-ADP potency, as MUFA and omega-3 PUFA present in such polar lipids usually are released intracellularly by the activity of a membrane phospholipase A2 (PLA2) enzyme, and then they can either antagonistically inhibit cycloxigenase to produce eicosanoids (one of PAF’s and the main of ADP’s pathways of platelet activation and aggregation), or they can influence the expression of several anti-inflammatory genes and enzymes by binding to and activating specific transcription factors for specific genes that promote reduction in inflammation, thrombosis and oxidative stress [3,4].

This observation also seems to explain the differences between the non-fermented and the fermented products in terms of the anti-PAF and anti-ADP potential of their TAC extracts. More specifically, as shown in the proposed structures of the PC and PE in both Table 4 and Table 5 for the non-fermented soy and oat products, the fatty acid present in their *sn*-2 position is usually a saturated one and mainly the omega-6 PUFA fatty acid linoleic acid (*n*-6 18:2 PUFA) and at some point, a MUFA one like the 16:1 or 18:1 ones. In contrast, and as shown in Table 4 and Table 5, in the fermented products, the proposed PL structures for both PE and PC and in oat and soy yogurt-style products show that the fatty acid at the *sn*-2 position of is usually either a MUFA (e.g., 16:1, 18:1) or the alpha linolenic fatty acid (*n*-3 18:3). According to the above possess, these fatty acids possess pleiotropic bioactivities when released intracellularly by PLA2 [3,4], which seems to explain the stronger anti-PAF and anti-ADP effects observed in the TAC from the fermented products compared to the activities observed in the TAC of the non-fermented products. Similar better outcomes for the TAC extracts of the fermented dairy alternatives versus the TAC from non-fermented ones have also been previously observed in dairy alternatives from rice and almond, and to a lesser extent in those from coconut [14].

Collectively, all the above support that in the present study, a structure–activity relationship has been obtained for these differences, which was not previously achieved. Overall, our LC–MS analysis revealed that fermentation modified the fatty acid composition at the *sn*-2 position of phosphatidylcholine (PC) and phosphatidylethanolamine (PE) molecules, shifting from predominantly omega-6 fatty acids (e.g., linoleic acid) in non-fermented products to a higher proportion of monounsaturated fatty acids and omega-3 polyunsaturated fatty acids (e.g., α-linolenic acid) in fermented products. These fatty acids may contribute to enhanced biological activity through several mechanisms:

- Structural mimicry of platelet-activating factor (PAF) in ether-linked phospholipids, allowing these molecules to act as competitive antagonists at the PAF receptor.
- Release of MUFA and *n*-3 PUFA via phospholipase A_2_ activity, which may subsequently modulate inflammatory signaling pathways (inhibition of intracellular COX-2, modulation of gene expression towards anti-inflammatory and inflammation-resolving conditions, etc.).
- Modulation of eicosanoid biosynthesis, since omega-3 fatty acids compete with omega-6 substrates in cyclooxygenase and lipoxygenase pathways, towards more inflammation-resolving compounds (i.e., resolvins from *n*-3 PUFA) rather than inflammatory eicosanoids (from *n*-6 PUFA).

Collectively, these structural modifications of the polar lipid content may explain the stronger anti-PAF and antiplatelet activities observed in the PL of the TAC extracts of fermented products versus the unfermented ones.

Nevertheless, beyond compositional aspects, accumulated evidence highlights the physiological importance of these phospholipids. PEs are integral components of intestinal epithelial cells and mucus layers, where they contribute to barrier integrity, regulation of goblet cell differentiation, and protection against apoptosis, thereby supporting intestinal morphology and function [48]. PCs, in turn, play essential roles in hepatic physiology as major constituents of hepatocyte membranes and as critical mediators of very-low-density lipoprotein (VLDL) assembly and triglyceride secretion, while both PC and PE beneficially affect HDL functionality [49].

PC deficiency has been associated with alleviating oxidative stress, inflammation, and progressive liver pathology. Furthermore, adequate PC intake, and especially intake of those PCs that can inhibit PAF and other thrombo-inflammatory mediators strongly, has been linked to anti-inflammatory and antithrombotic cardioprotective effects, modulation of blood pressure, and potential neuroprotective benefits, including support of cognitive function [3,4,50]. Emerging evidence also suggests associations between phosphatidylcholine intake and reduced risk of certain malignancies [4].

Taken together, the present findings, in conjunction with the existing literature, indicate that both fermented and non-fermented soy- and oat-based products are relevant dietary sources of bioactive phosphatidylethanolamines (PEs) and phosphatidylcholines (PCs). Their presence in the amphiphilic TAC fractions of extracts from these sources may contribute to the anti-inflammatory and antithrombotic properties observed in this study, supporting the potential role of these plant-based foods in promoting cardiovascular, hepatic, intestinal, and possibly neurological health.

## 4. Conclusions

This study demonstrates that fermentation significantly enhances the biofunctional properties of soy- and oat-based dairy alternatives. Fermented products exhibited improved antioxidant capacity and stronger anti-inflammatory and antiplatelet activities, particularly within the amphiphilic lipid fractions. Structural analysis revealed that these effects are associated with changes in the polar lipid profile and fatty acid composition, including favorable modifications of the *n*-6/*n*-3 fatty acid ratio and the presence of bioactive phospholipid species.

Overall, the findings highlight fermentation as an effective technological approach to improve the functional value of plant-based dairy alternatives. Fermented soy and oat yogurt-type products may therefore represent promising dietary sources of bioactive lipids with potential relevance for the modulation of thrombo-inflammatory processes associated with cardiometabolic diseases.

However, the present findings are based on in vitro and ex vivo assays. Future studies integrating targeted lipidomics, bioavailability investigations, and clinical trials are needed to further clarify the mechanisms involved and confirm the health relevance of these bioactive compounds in humans.

## Figures and Tables

**Table 1:** Extraction yield of total lipids (TL), total lipophilic compounds (TLC), and total amphiphilic compounds (TAC), expressed in g/100 g sample (mean ± SD, *n* = 3) in the four different product types.

Samples	TAC	TLC	TL
Soy drink	2.42 ± 1.26	1.42 ± 0.41	3.84 ± 1.27
Oat drink	3.32 ± 1.84	0.32 ± 0.04	3.64 ± 1.82
Soy yogurt	4.23 ± 1.66	0.23 ± 0.10	4.46 ± 1.74
Oat yogurt	4.53 ± 1.48	1.78 ± 0.29	6.31 ± 1.29

**Table 2:** The fatty acid profile of the polar lipids in the TAC extracts for each lipid extract from soy and oat beverage and yogurt samples after saponification, expressed for each fatty acid (FA) as a percentage composition of the total fatty acids in each sample evaluated (mean ± standard deviation (SD), *n* = 3).

Fatty Acid	Soy Drink	Soy Yogurt	Oat Drink	Oat Yogurt
C8:0	1.07 ± 0.02	1.11 ± 0.03	0.81 ± 0.01	1.1 ± 0.01
C9:0	0.08 ± 0.01	0.08 ± 0.01	0.10 ± 0.01	0.1 ± 0.01
C10:0	1.40 ± 0.01	1.44 ± 0.02	1.10 ± 0.01	1.46 ± 0.01
C11:0	0.02 ± 0.01	0.02 ± 0.01	0.02 ± 0.01	0.02 ± 0.01
C12:0	5.38 ± 0.02	5.62 ± 0.07	4.31 ± 0.04	5.63 ± 0.03
C13:0	0.03 ± 0.01	0.03 ± 0.01	0.03 ± 0.01	0.03 ± 0.01
C14:0	1.92 ± 0.01	2.03 ± 0.06	1.74 ± 0.01	2.02 ± 0.01
C15:0	0.36 ± 0.01	0.38 ± 0.01	0.34 ± 0.01	0.37 ± 0.01
C16:0	33.98 ± 0.08	36.24 ± 0.2	35.32 ± 0.07	35.52 ± 0.08
C16:1	0.19 ± 0.01	0.20 ± 0.01	0.26 ± 0.01	0.19 ± 0.01
C17:0	0.87 ± 0.01	0.97 ± 0.04	0.86 ± 0.03	0.92 ± 0.01
C18:0	44.65 ± 0.16	48.15 ± 0.71	40.84 ± 0.19	48.07 ± 0.1
C18:1	3.78 ± 0.04	2.0 ± 0.14	8.36 ± 0.05	2.65 ± 0.04
C18:2	4.73 ± 0.03	0.54 ± 0.02	4.75 ± 0.07	0.76 ± 0.01
C18:3	0.63 ± 0.01	0.23 ± 0.01	0.25 ± 0.01	0.26 ± 0.01
C19:0	0.06 ± 0.01	0.06 ± 0.01	0.06 ± 0.01	0.07 ± 0.01
C20:0	0.77 ± 0.04	0.83 ± 0.1	0.75 ± 0.01	0.76 ± 0.06
C20:1	0.07 ± 0.01	0.04 ± 0.03	0.11 ± 0.01	0.07 ± 0.01
C20:5	0.01 ± 0.01	0.01 ± 0.01	0.01 ± 0.01	0.01 ± 0.01
SFA	90.60 ± 0.05	96.98 ± 0.20	86.26 ± 0.03	96.06 ± 0.04
UFA	9.40 ± 0.05	3.02 ± 0.20	13.74 ± 0.03	3.94 ± 0.04
MUFA	4.04 ± 0.05	2.25 ± 0.18	8.73 ± 0.05	2.91 ± 0.01
PUFA	5.37 ± 0.03	0.77 ± 0.02	5.01 ± 0.07	1.03 ± 0.01
*n*-6 PUFA	4.73 ± 0.03	0.54 ± 0.02	4.75 ± 0.07	0.76 ± 0.01
*n*-3 PUFA	0.64 ± 0.01	0.24 ± 0.01	0.26 ± 0.01	0.27 ± 0.01
*n*-6/*n*-3 PUFA	7.42 ± 0.12	2.24 ± 0.09	18.58 ± 0.53	2.82 ± 0.07

SFA = saturated fatty acids; UFA = unsaturated fatty acids; MUFA = monounsaturated fatty acids; PUFA = polyunsaturated fatty acids; *n*-6 = omega-6; *n*-3 = omega-3.

**Table 3:** The free fatty acid profile of TAC extracts for each lipid extract from soy and oat beverage and yogurt samples prior to saponification is expressed for each fatty acid (FA) as a percentage composition of the total fatty acids in each sample evaluated (mean ± standard deviation (SD), *n* = 3).

Fatty Acid	Soy Drink	Soy Yogurt	Oat Drink	Oat Yogurt
C8:0	0.19 ± 0.01	0.13 ± 0.01	0.19 ± 0.01	0.13 ± 0.01
C9:0	0.38 ± 0.03	0.34 ± 0.01	0.38 ± 0.01	0.34 ± 0.03
C12:0	0.41 ± 0.10	0.23 ± 0.10	0.21 ± 0.06	0.20 ± 0.05
C14:0	2.15 ± 0.46	1.93 ± 0.30	1.75 ± 0.15	1.63 ± 0.13
C15:0	1.40 ± 0.21	1.42 ± 0.11	1.18 ± 0.02	1.22 ± 0.01
C16:0	24.33 ± 1.11	23.87 ± 0.33	27.37 ± 0.74	24.57 ± 2.37
C16:1	1.57 ± 0.08	1.72 ± 0.04	1.46 ± 0.05	1.45 ± 0.10
C17:0	3.44 ± 0.10	3.66 ± 0.06	2.65 ± 0.16	3.00 ± 0.18
C18:0	55.77 ± 0.43	53.88 ± 0.31	44.64 ± 1.30	52.15 ± 2.16
C18:1	5.62 ± 0.13	6.19 ± 0.47	12.11 ± 0.30	7.92 ± 0.61
C18:2	5.45 ± 0.07	1.60 ± 0.03	4.13 ± 0.09	1.47 ± 0.10
C18:3	2.08 ± 0.05	1.60 ± 0.04	3.49 ± 0.11	5.40 ± 0.14
C20:1	0.58 ± 0.06	0.62 ± 0.02	0.44 ± 0.03	0.53 ± 0.04
SFA	88.50 ± 0.12	85.46 ± 0.44	78.37 ± 0.54	83.23 ± 0.73
UFA	11.50 ± 0.12	11.73 ± 0.42	21.63 ± 0.54	16.77 ± 0.73
MUFA	7.60 ± 0.09	8.53 ± 0.43	14.01 ± 0.33	9.90 ± 0.70
PUFA	3.90 ± 0.11	3.21 ± 0.04	7.62 ± 0.21	6.87 ± 0.08
*n*-6 PUFA	2.08 ± 0.07	1.60 ± 0.03	4.13 ± 0.09	1.47 ± 0.10
*n*-3 PUFA	1.81 ± 0.05	1.60 ± 0.04	3.49 ± 0.11	5.40 ± 0.14
*n*-6/*n*-3 PUFA	1.15 ± 0.02	1.00 ± 0.03	1.18 ± 0.01	0.27 ± 0.02

SFA = saturated fatty acids; UFA = unsaturated fatty acids; MUFA = monounsaturated fatty acids; PUFA = polyunsaturated fatty acids; *n*-6 = omega-6; *n*-3 = omega-3.

**Table 4:** Summary of the main classes of polar lipids present in oat products.

Main Classes of PL	Mr	TAC Extracts from Oat Milk	Proposed Structures
		Representative Molecular Species	
PEs	703.1859	PE O-34:1	[i.e., PE O-16:0/18:1 or PE O-18:0/16:1]
	705.2031	PE O-34:0	[i.e., PE O-16:0/18:0]
	725.3959	PE O-36:4	[i.e., PE O-18:2/18:2]
	729.1441	PE O-36:2	[i.e., PE O-18:0/18:2]
PCs	703.1859	PC O-32:1	[i.e., PC O-16:0/16:1]
	705.2031	PC O-32:0	[i.e., PC O-16:0/16:0]
	729.1441	PC O-34:2	[i.e., PC O-16:0/18:2]
	731.1642	PC O-34:1	[i.e., PC O-16:0/18:1]
	733.2346	PC O-34:0	[i.e., PC O-16:0/18:0]
	753.1884	PC O-36:4	[i.e., PC O-18:2/18:2]
	759.2506	PC O-36:1	[i.e., PC O-18:0/18:1]
	761.2656	PC O-36:0	[i.e., PC 18:0/18:0]
	787.1521	PC O-38:1	i.e., PC O-20:0/18:1]
	789.444	PC O-38:0	[i.e., PC O-20:0/18:0]
		**TAC Extracts** **from Oat Yogurt**	
PEs	725.4915	PE O-36:4	[i.e., PE O-18:1/18:3]
	729.1437	PE O-36:2	[i.e., PE O-18:1/18:1]
	731.2191	PE O-36:1	[i.e., PE O-18:0/18:1]
	733.2346	PE O-36:0	[i.e., PE 18:0/18:0]
	777.4078	PE O-38:0;O	[i.e., PE 20:0/18:0]
PCs	729.1437	PC O-34:2	[i.e., PC O-16:1/18:1]
	731.2191	PC O-34:1	[i.e., PC O-16:0/18:1]
	733.2346	PC O-34:0	[i.e., PC O-16:0/18:0]
	753.5231	PC O-36:4	[i.e., PC O-18:1/18:3]
	761.2655	PC O-36:0	[i.e., PC O-18:0/18:0]

**Table 5:** Summary of the main classes of polar lipids present in soy products.

Main Classes of PL	Mr	TAC Extracts from Soy Milk	Proposed Structures
		Representative Molecular Species	
PEs	729.1438	PE O-36:2	[i.e., PE O-18:0/18:2]
	733.2343	PE O-36:0	[i.e., PE O-18:0/18:0]
	757.405	PE O-38:2	[i.e., PE O-20:0/18:2]
	761.2655	PE O-38:0	[i.e., PE O-18:0/20:0]
PCs	646.3329	PC O-34:2	[i.e., PC O-16:0/18:2]
	658.508	PC O-36:4	[i.e., PC O-18:1/18:3]
	743.1472	PC O-36:5	[i.e., PC O-18:2/18:3]
	747.2498	PC O-32:0	[i.e., PC O-16:0/16:0]
	753.5227	PC O-36:4	[i.e., PC O-18:1/18:3]
	761.2655	PC O-36:0	[i.e., PE O-18:0/18:0]
		**TAC Extracts** **from Soy Yogurt**	
PEs	675.1604	PE O-32:1	[i.e., PE O-16:0/16:1]
	705.2034	PE O-34:0	[i.e., PE O-16:0/18:0]
	725.4915	PE O-36:4	[i.e., PE O-18:1/18:3]
	729.1441	PE O-36:2	[i.e., PE O-18:1/18:1]
	777.4073	PE O-38:0;O	[i.e., PE O-20:0/18:0]
PCs	580.9632	PC O-34:0	[i.e., PC O-16:0/18:0]
	677.1711	PC O-36:0	[i.e., PC O-18:0/18:0]
	705.2034	PC O-38:1	[i.e., PC O-20:0/18:1]
	729.1441	PC O-32:0	[i.e., PC O-16:0/16:0]

## Data Availability

All raw data supporting the conclusions of this article are embedded in the article and its Supplementary Materials, while any other raw data on bulky files of chromatograms and LC-MS analysis can be made available by the project PI and the corresponding author (A.T.) upon request.

## Supplementary Materials

The following supporting information can be downloaded at: https://www.mdpi.com/article/10.3390/nu18081260/s1. Figure S1: ATR-FTIR spectrum of standard substances, GA: gallic acid, quercetin: quercetin, catechin: catechin, b-car: b-carotene, polar lipids: polar soybean lipids. (The Y-axis is %Transmittance and the X-axis is cm^−1^).

## Abbreviations

ABTS	2,2′-Azino-bis(3-ethylbenzothiazoline-6-sulfonic acid)
DPPH	2,2-Diphenyl-1-picrylhydrazyl
FA	Fatty Acids
FRAP	Ferric Reducing Antioxidant Power
GAE	Gallic Acid Equivalents
hPRP	Human Platelet-Rich Plasma
IC_50_	Half Maximal Inhibitory Concentration
LC–MS	Liquid Chromatography–Mass Spectrometry
*n*-3	Omega-3 Fatty Acids
*n*-6	Omega-6 Fatty Acids
PAF	Platelet-Activating Factor
PC	Phosphatidylcholine
PE	Phosphatidylethanolamine
PL	Polar Lipids
TAC	Total Amphiphilic Compounds
TLC	Total Lipophilic Compounds
*S. thermophilus*	*Streptococcus thermophilus*
*G. max*	*Glycine max*
*A. sativa*	*Avena sativa*
