# Supplementary material for: Fermentation Enhances Antioxidant, Antiplatelet, and Anti-Inflammatory Properties of Oat- and Soy-Derived Dairy Alternatives
Source: Nutrients. 2026 Apr 16;18(8):1260. doi: 10.3390/nu18081260 (PMC13119166; doi:10.3390/nu18081260)
Supplement: Supplementary file 1 [file nutrients-18-01260-s001.zip › nutrients-4192410-supplementary.pdf]

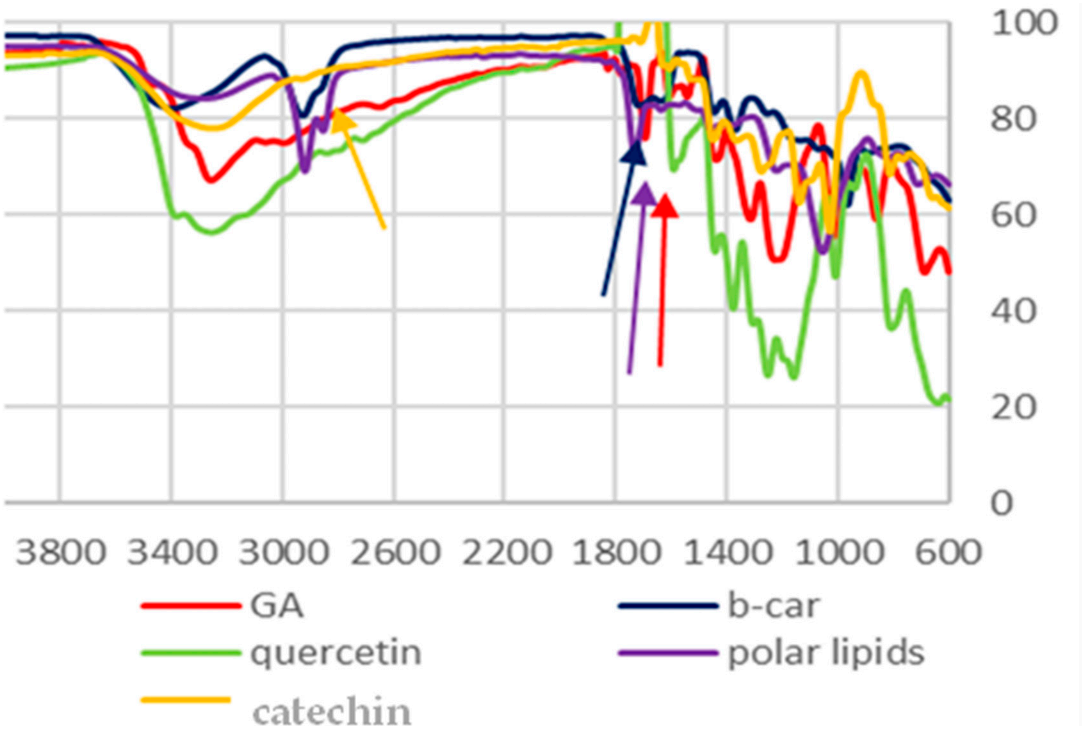

Supplementary Figure S1. ATR-FTIR spectrum of standard substances, GA: gallic acid, quercetin: quercetin, catechin: catechin, b-car: b-carotene, polar lipids: polar soybean lipids. (The Y-axis is %Transmittance and the X-axis is cm^{-1}).
